# Neural stem cell delivery via porous collagen scaffolds promotes neuronal differentiation and locomotion recovery in spinal cord injury

**DOI:** 10.1038/s41536-020-0097-0

**Published:** 2020-06-15

**Authors:** Alexandra Kourgiantaki, Dimitrios S. Tzeranis, Kanelina Karali, Konstantina Georgelou, Efstathia Bampoula, Sotirios Psilodimitrakopoulos, Ioannis V. Yannas, Emmanuel Stratakis, Kyriaki Sidiropoulou, Ioannis Charalampopoulos, Achille Gravanis

**Affiliations:** 1 Department of Pharmacology, School of Medicine, University of Crete, Heraklion, 71003 Greece; 2 Institute of Molecular Biology and Biotechnology, Foundation for Research and Technology–Hellas, Heraklion, 71003 Greece; 3 Department of Mechanical Engineering, Massachusetts Institute of Technology, Cambridge, MA 02139 USA; 4 Department of Biology, University of Crete, Heraklion, 71003 Greece; 5 Institute of Electronic Structure and Laser, Foundation for Research and Technology–Hellas, Heraklion, 71003 Greece

**Keywords:** Neural stem cells, Spinal cord injury, Translational research

## Abstract

Neural stem cell (NSC) grafts have demonstrated significant effects in animal models of spinal cord injury (SCI), yet their clinical translation remains challenging. Significant evidence suggests that the supporting matrix of NSC grafts has a crucial role in regulating NSC effects. Here we demonstrate that grafts based on porous collagen-based scaffolds (PCSs), similar to biomaterials utilized clinically in induced regeneration, can deliver and protect embryonic NSCs at SCI sites, leading to significant improvement in locomotion recovery in an experimental mouse SCI model, so that 12 weeks post-injury locomotion performance of implanted animals does not statistically differ from that of uninjured control animals. NSC-seeded PCS grafts can modulate key processes required to induce regeneration in SCI lesions including enhancing NSC neuronal differentiation and functional integration in vivo, enabling robust axonal elongation, and reducing astrogliosis. Our findings suggest that the efficacy and translational potential of emerging NSC-based SCI therapies could be enhanced by delivering NSC via scaffolds derived from well-characterized clinically proven PCS.

## Introduction

Traumatic spinal cord injury (SCI) results in devastating disabilities that affect millions of patients worldwide. Current clinical treatment of SCI is limited to surgical intervention for spinal cord decompression^1^ and the delivery of methylprednisolone, a corticosteroid of controversial efficacy^2^. The development of effective SCI treatments is still an unmet clinical need, as no treatment has demonstrated consistent efficiency and safety^3^.

The multifactorial, complex nature of SCI^4^ motivated the development of combinatorial treatments that would integrate the effects of diffusible factors (small molecules, neurotrophic factors, and biologics^5,6^), cell therapies, and biomaterials. In particular, cell-based treatments utilize stem cells to replace cells lost during SCI and enhance axonal elongation and synaptic plasticity^7^. Among several types of stem cells evaluated, neural stem cells (NSCs) are of particular interest, as they can differentiate to both neurons and glia, secrete neurotrophic factors, and their application does not suffer from the safety concerns of pluripotent stem cells^8^. Indeed, several NSC-based grafts have provided encouraging results in rodent and primate SCI models^9–12^.

However, the clinical translation of NSC treatments remains challenging. The first phase I clinical trial of a human NSC cell line in chronic SCI patients reported encouraging safety results but very limited efficacy^13^, possibly due to NSC delivery in suspension. Indeed, several studies have demonstrated that NSC delivery at SCI sites without a supporting matrix results in extensive cell death, poor neuronal differentiation, and suspension leak away from the lesion site^14,15^. Several of these obstacles can be overcome by delivering NSCs inside biomaterials. Biomaterials can enhance NSC therapies by localizing cell delivery at the injury site, filling the lesion cavity volume, protecting delivered cells from immune attack, and enhancing axonal elongation by providing adhesion cues and downregulating deleterious inflammation and scar formation^16–20^. Although certain NSC grafts have utilized biomaterials (e.g., fibrin hydrogels, collagen gels), none of them has received regulatory approval before^9–11,21^.

On the other hand, porous collagen-based scaffolds (PCSs) have revolutionized the clinical treatment of severe injuries in skin and peripheral nerves. However, 40 years after the discovery of their ability to induce dermis regeneration^22^, PCS have not managed to impact the clinical treatment of central nervous system (CNS) injuries, including SCI. Cell-free PCS grafts applied to full-transection rat SCI models managed to reduce and favorably align the resulting collagenous scar tissue and reduce cyst cavity formation at the lesion site^16,19^. In the same rat transection SCI model, NSC-seeded PCS grafts did not induce significant functional improvement compared with cell-free grafts, possibly due to the difficult-to-reverse nature of full-transection SCI or the use of adult (instead of embryonic) NSCs. Despite the large number of studies that report effects on NSCs by various biomaterials, including a few that utilized porous scaffolds (in this work, the term “scaffold” refers to a porous biomaterial fabricated in dry state, therefore excludes hydrogels)^23,24^, these reports usually have lack detailed characterization of the biomaterial used (e.g., structure, in-vivo degradation, surface chemistry) and cannot be used to draw conclusions on the specific biomaterial features that have significant regenerative effects in SCI in vivo. No prior study has described in detail the effects of PCS on potent NSCs and the ability of PCS to provide NSCs an appropriate support matrix at SCI sites. Motivated by the in-vivo effects of fibrin hydrogels to NSCs^9–11^, and by the ability of keratinocyte-seeded Dermis Regeneration Template to accelerate epidermis formation in large skin wounds^22,25^, here we evaluate whether PCS grafts could host NSCs and leverage the regenerative potential of NSCs in SCI in vivo.

The present study demonstrates that grafts based on well-characterized PCS^26,27^, similar to Food and Drug Administration (FDA)-approved scaffolds utilized in regenerative medicine^22,25,28^, can deliver and protect embryonic NSCs at SCI sites, leading to a statistically significant improvement in locomotion recovery in a mouse dorsal column crush SCI model, so that 12 weeks post-injury locomotion performance was not statistically different than uninjured animals. Extensive in vitro and in-vivo data demonstrate the ability of PCS grafts to modulate key processes required to induce regeneration in CNS lesions. PCS grafts protected NSCs and enabled NSC neural differentiation and functional integration in vivo, facilitated axonal elongation at the lesion site and through the lesion boundary, enabled NSC migration into the surrounding tissue, and reduced astrogliosis. Our findings suggest the development of a new generation of SCI grafts that will deliver NSCs via optimized scaffolds derived from well-characterized clinically proven PCS.

## Results

### Establishment of 3D culture of NSCs inside PCSs in vitro

**Fig. 1:**
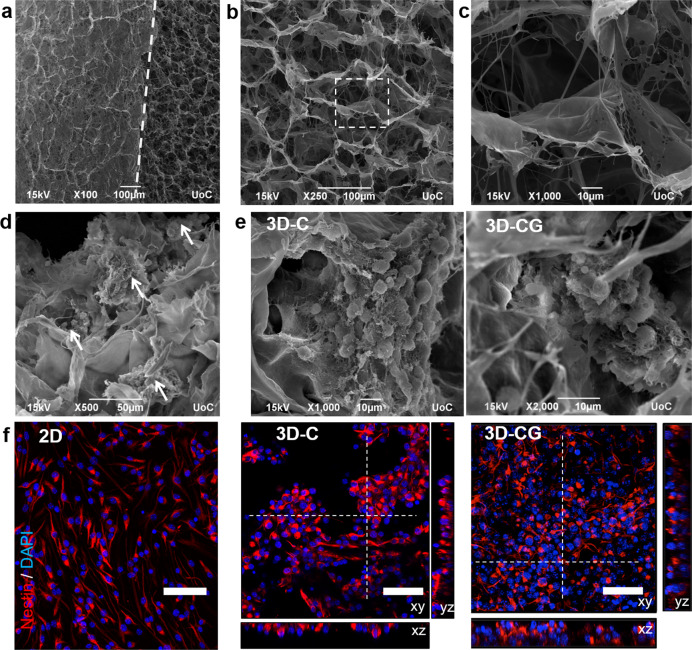
3D culture of mouse embryonic neural stem cells inside porous collagen-based scaffolds. **a** SEM image highlighting the interface (dashed line) between the scaffold surface (left) and the scaffold interior (right). **b** SEM image of a porous collagen-GAG scaffold highlighting its porous structure. **c** High-magnification image of the region shown in **b**. **d** SEM image of neurospheres (white arrows) grown inside a collagen-GAG (3D-CG) scaffold at 3 DIV. **e** SEM images of neurospheres grown inside collagen (3D-C) or collagen-GAG (3D-CG) scaffolds at 3 DIV. **f** Representative confocal fluorescence images of nestin^+^ NSCs (red) grown on a PDL-laminin-coated coverslip (2D), inside a collagen scaffold (3D-C) or inside a collagen-GAG scaffold (3D-CG) at 3 DIV. Scale bars, 50 μm.

To study PCS–NSC interactions and prepare PCS grafts for implantation at SCI sites, a three-dimensional (3D) culture system based on PCS was established. NSCs were seeded and studied inside cylindrical samples of collagen scaffolds (denoted as 3D-C) similar to grafts utilized in peripheral nerve regeneration^27^, or inside samples of porous collagen-glycosaminoglycan (GAG) scaffolds (denoted as 3D-CG) similar to grafts utilized in skin regeneration^22,25^. Compared with 3D-C, 3D-CG scaffolds contain chondroitin-6-sulfate (Ch6S). Both the PCS types were fabricated using identical protocols, leading to sponges of 0.5% mass fraction, 3-week in-vivo degradation half-life, and ≈95 μm mean pore diameter (Fig. 1a-c)^26,27^. NSCs were seeded in single-cell suspension and easily infiltrated inside PCS pores, where they adhered to PCS struts as single cells (Fig. 1d, f) or formed neurosphere-like aggregates (Fig. 1e, f). After 3 days in vitro (DIV), >99% of cells stained for nestin (Fig. 1f). NSC activity did not cause detectable scaffold degradation after 10 DIV, suggesting that once seeded in PCS, NSCs can be manipulated for several days before grafting the resulting tissue construct into an SCI site.

### Regulation of NSC proliferation, survival, and differentiation by PCSs in vitro

**Fig. 2:**
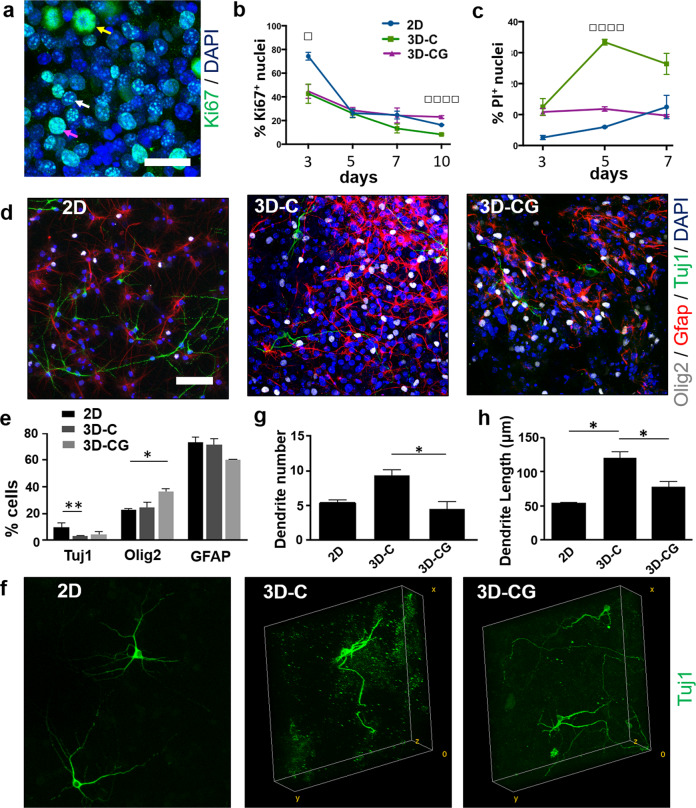
Regulation of NSC proliferation, viability, and differentiation by PCS composition in vitro. **a** Fluorescence image of Ki67^+^ NSCs inside a porous collagen-GAG scaffold at 3 DIV. Arrows highlight cells at the early S phase (white arrow), the late S phase (purple arrow), or mitosis (yellow arrow). Scale bar, 30 μm. **b** Fraction of Ki67^+^ nuclei in the three NSC culture groups at 1, 3, 5, 7, and 10 DIV (*n* = 3). **c** Fraction of PI^+^ nuclei in the three NSC culture groups at 1, 3, 5, and 7 DIV (*n* = 3). **d** Representative confocal images of cells immunostained for Tuj1 (neurons; green), Olig2 (oligodendrocytes; gray), and Glial fibrillary acidic protein (GFAP, astrocytes; red) in 2D, 3D-C, and 3D-CG cultures at 7 DIV. Scale bar, 50 μm. **e** Fractions of Tuj1^+^, Olig2^+^, and GFAP^+^ cells in the three cell culture groups at 7 DIV. **f** 3D reconstruction of confocal *z*-stacks of NSC-derived Tuj1^+^ neurons growing on laminin-coated coverslips (2D) or inside porous scaffolds (3D-C, 3D-CG) at 7 DIV. Box size: 263 × 263 × 80 μm. **g** Number of dendrites per Tuj1^+^ neuron in the three NSC culture groups (*n* = 30). **h** Mean dendrite length per Tuj1^+^ neuron in the three NSC culture groups (*n* = 30). Results are expressed as mean ± SEM. *P < 0.05, **P < 0.01, ^□^*P*_1-way-ANOVA_ < 0.05, ^□□□□^*P*_1-way-ANOVA_ < 0.001.

The effect of PCS composition on NSC proliferation and viability was evaluated by quantifying the fraction of nuclei that stain positively for Ki67 and propidium iodine (PI), respectively, in NSCs grown inside PCS or on poly-d-lysine (PDL)-laminin-coated coverslips (two-dimensional (2D) control). The fraction of Ki67^+^ nuclei decreased progressively between 3 DIV and 10 DIV (Fig. 2a, b and Supplementary Fig. 1a). At 3 DIV, the fraction of Ki67^+^ nuclei was significantly higher in NSCs grown on 2D (2D: 74.3 ± 3.3%, *n* = 3; 3D-C: 42.7 ± 8.1%, *n* = 3, *P* < 0.05; 3D-CG: 44.7 ± 5.9%, *n* = 3, *P* < 0.05; *P*_1-way-ANOVA_ = 0.0187). At 10 DIV, the fraction of Ki67^+^ nuclei was significantly larger in 3D-CG culture (3D-CG: 23.0 ± 1.2%, *n* = 3; 3D-C: 8.3 ± 0.6%, *n* = 3; 2D: 16.3 ± 0.3%, *n* = 3; *P*_1-way-ANOVA_ < 0.0001). The fraction of PI^+^ nuclei at 3 DIV was similar in all groups and progressively increased between 3 DIV and 7 DIV (Fig. 2c and Supplementary Fig. 1b). The effect of PCS composition on the fraction of PI^+^ nuclei became statistically significant at 5 DIV (3D-CG: 33.4 ± 0.9%, *n* = 3; 3D-C: 10.8 ± 1.2%, *n* = 3, *P* < 0.01; 2D: 6.0 ± 0.1%, *n* = 3, *P* < 0.01; *P*_1-way-ANOVA_ = 0.0002). Overall, growing NSCs on laminin-coated coverslips or inside collagen-GAG scaffolds favored cell survival and proliferation compared with growing NSCs inside collagen scaffolds.

The effect of PCS chemical composition on NSC differentiation was investigated by growing NSCs inside porous scaffolds or on PDL-laminin-coated coverslips (2D culture control) for 3 days in complete medium and then 4 more days in a differentiation medium formulation that supports simultaneous NSC differentiation towards neurons, astrocytes, and oligodendrocytes. At 7 DIV, NSCs had differentiated mainly towards Glial fibrillary acidic protein (GFAP)^+^ cells (glial-restricted progenitors and astrocytes) and less towards Olig2^+^ (oligodendrocyte lineage) cells or Tuj1^+^ neurons (Fig. 2d, e), in agreement with previous reports^29^. NSC differentiation in 2D culture resulted in a larger fraction of Tuj1^+^ neurons than that of 3D-C culture (2D: 9.8 ± 0.4%, *n* = 3; 3D-C: 3.2 ± 0.14%, *n* = 3, *P* = 0.0054). NSC differentiation in 3D-CG resulted in significantly larger fraction of Olig2^+^ cells (3D-CG: 36.3 ± 2.3%, *n* = 3; 2D: 23.0 ± 0.5%, *n* = 3, *P* = 0.0319).

### Regulation of neuronal differentiation by PCSs in vitro

Maturation of NSC-derived neurons was initially evaluated by quantifying two morphological features (neurite branching, mean length of neuron dendrites) of Tuj1^+^ neurons at 7 DIV (Fig. 2f-h). Interestingly, NSC-derived neurons in 3D-C culture exhibited significantly higher axonal branching (3D-C: 9.3 ± 0.8, *n* = 27; 3D-CG: 4.5 ± 0.9, *n* = 20; *P* = 0.0364; Fig. 2g) and sustained significantly longer neuronal dendrites (3D-C: 120.3 ± 9.2 μm, *n* = 27; 3D-CG: 78.3 ± 7.2 μm, *n* = 20; *P* = 0.0377; Fig. 2h) compared with NSC-derived neurons in 3D-CG culture.

**Fig. 3:**
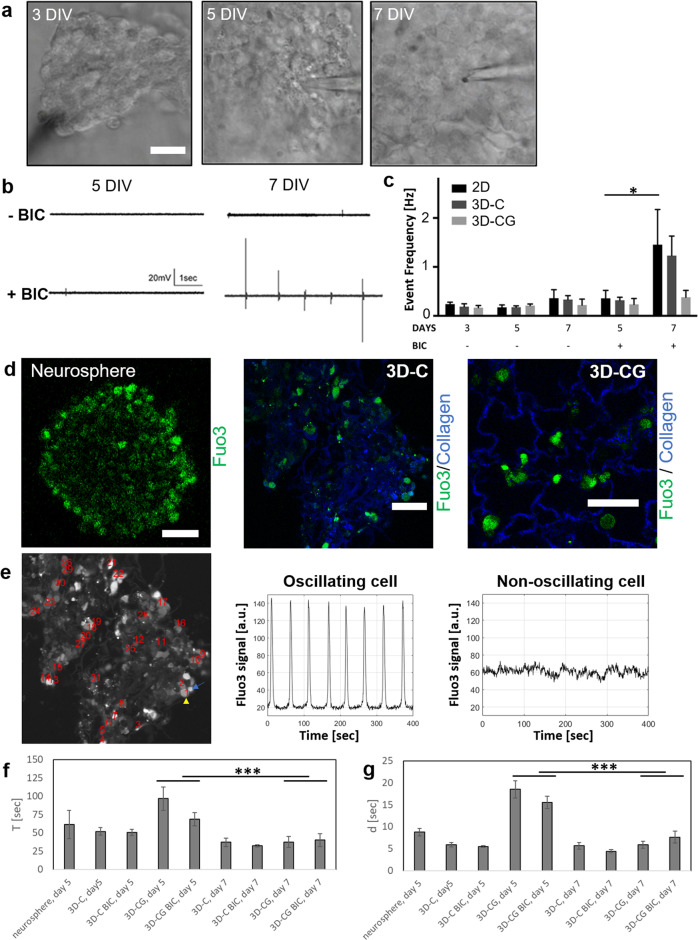
Porous collagen scaffolds enhance NSC neural differentiation in vitro. **a** Representative bright-field microscopy images of neurospheres grown in PCS during electrophysiological recordings at 3, 5, and 7 DIV. Scale bar, 20 μm. **b** Representative field recordings of spontaneous activity acquired in neurospheres grown inside collagen scaffolds (3D-C) before (top row) and after (bottom row) bicuculline treatment, at 5 and 7 DIV. **c** Quantification of spontaneous activity event frequency in neurospheres grown in 2D or 3D cultures at 3, 5, and 7 DIV (*n* = 6). **d** Representative fluorescent image of Fluo3 emission (green) in neurospheres grown in suspension or NSCs seeded in collagen or collagen-GAG scaffolds (blue) at 5 DIV. Scale bars, 50 μm. **e** Representative time profiles of Ca^+2^-induced Fluo3 signal in single cells grown inside a porous collagen scaffold. Comparison of profiles in two neighboring cells, one of which displays Fluo3 oscillation (yellow arrowhead) and one does not (cyan arrow). **f**, **g** Quantification of Ca^+2^ oscillations: mean period *T* between spontaneous events (**f**) and FWHM *d* of event duration (**g**) in NSC grown inside collagen or collagen-GAG scaffolds at 5 or 7 DIV, in the presence or absence of bicuculline (*n* = 3 to 5). Results are expressed as mean ± SEM. *P < 0.05, ***P < 0.001.

We further evaluated the maturation of NSC-derived neurons by quantifying their spontaneous activity. Field recordings revealed low level of spontaneous activity in neurospheres formed in 3D-C, 3D-CG cultures, and 2D (standard NSC neurosphere culture) at 3, 5, and 7 DIV (Fig. 3a). Given the significant role of GABA_A_ signaling in neuronal differentiation and maturation during embryonic neurogenesis^30^, we investigated whether bicuculline, a GABA_A_ antagonist, could affect spontaneous activity measurements. Although at 5 DIV no group was responsive to bicuculline, at 7 DIV neurospheres grown inside collagen scaffolds (3D-C) profoundly increased their spontaneous activity in response to bicuculline (5 DIV: 0.32 ± 0.06 events per second, *n* = 8; 7 DIV: 1.23 ± 0.04 events per second, *n* = 8; *P* < 0.05; Fig. 3b, c).

To quantify spontaneous activity in single cells located inside scaffolds, we utilized time-lapsed confocal fluorescent microscopy to monitor cytosolic Ca^+2^ fluctuations in fluo3-stained NSCs at 5 and 7 DIV. Single-cell analysis revealed that a fraction of cells exhibited periodic oscillations of cytosolic Ca^+2^, consisting of repeated Ca^+2^ events (Fig. 3d, e). Such oscillations were observed only in neurospheres or in cell-rich scaffold regions, and not on isolated cells, suggesting that cell–cell interactions were required for their generation. At 5 DIV, NSCs grown in collagen scaffolds exhibited more frequent (smaller period *T* between events) and sharper [smaller event full width at half maximum (FWHM) *d*] Ca^+2^ events compared with NSCs grown in collagen-CG scaffolds (Fig. 3f-g and Supplementary Video 1), indicative of more advanced neural maturation. Between 5 DIV and 7 DIV, both *T* and *d* decreased in both 3D-C and 3D-CG cell culture groups (*P* < 0.001). Multiple-factor analysis revealed a significant effect of scaffold type (*P* < 0.05) on both *T* and *d*. On the other hand, bicuculline treatment did not affect significantly duration d or period *T*.

### Porous collagen scaffolds support the formation of networks of functional neurons in vitro

We then evaluated the ability of collagen scaffolds to support the formation of networks of functional terminally differentiated neurons inside them, a process requiring significant axon elongation and synapse formation. Five days after seeding E13.5 mouse dorsal root ganglia (DRG) cells inside collagen scaffolds, cells had attached extensively on the scaffold and neurons had extended long neurites with synaptic activity along the scaffold surface (Supplementary Fig. 2a-c). At DIV 7, a 3D network of DRG neurons had formed inside the scaffold (Fig. 3g), consisting of long axons, and stained positive for neurofilament heavy chain and the presynaptic marker synaptophysin (Supplementary Fig. 2d, e). The ability of neuronal cells to elongate axons into collagen scaffolds was not limited to primary cells. Preliminary experiments suggest that spinal cord neural cells derived from mouse embryonic stem cells can adhere and grow axons through the pores of collagen scaffolds (Supplementary Fig. 2f).

### NSC-seeded collagen scaffolds significantly improved locomotion recovery after dorsal column crush SCI in mice

**Fig. 4:**
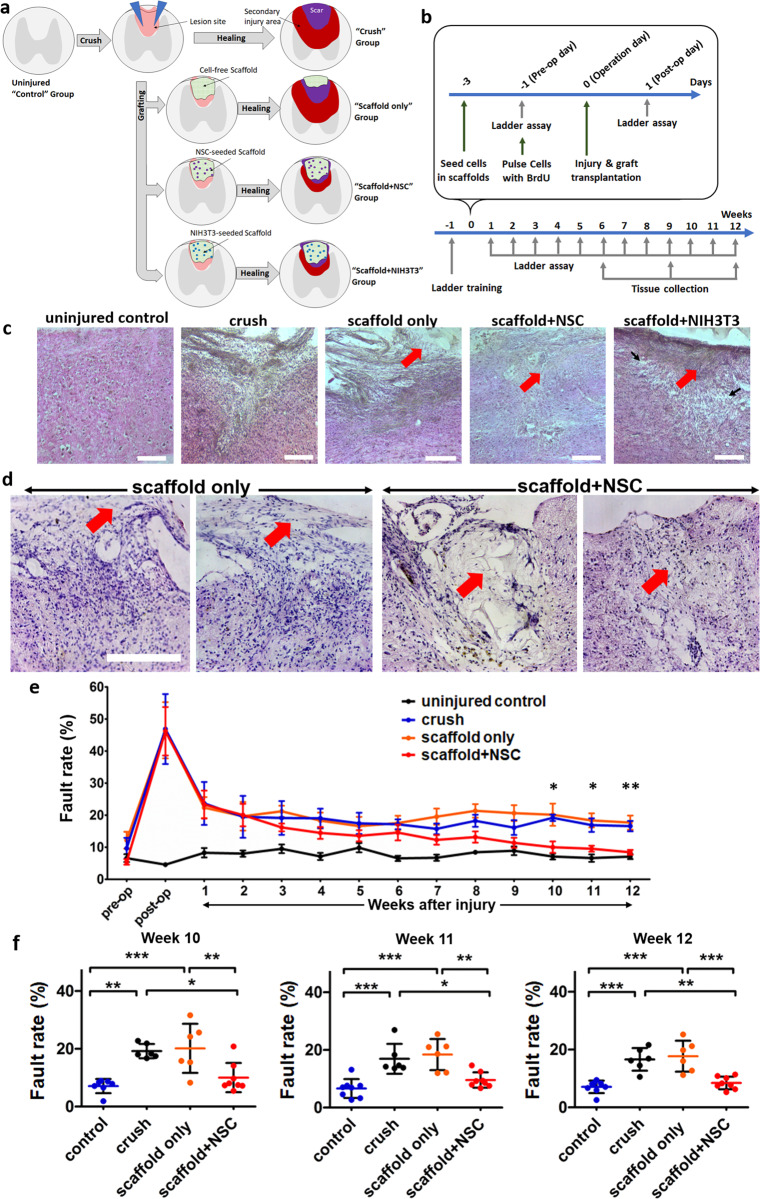
Porous collagen scaffolds seeded with embryonic NSCs enhanced locomotion recovery in a mouse dorsal column crush SCI model. **a** Schematic of the injury model and the three animal groups considered in this study. **b** Timeline of animal experiments, demonstrating BrdU labeling, injury and grafting, locomotion recovery quantification, and animal killing for histology. **c** H&E staining of spinal cord parasagittal sections from all animal groups 6 weeks post injury. Red arrows indicate residual scaffold and black arrows indicate areas of cavitation. Scale bars, 200 μm. **d** Higher-magnification images of spinal cord cross-sections stained by H&E from the scaffold-only and scaffold + NSC animal groups 6 weeks post injury. The location of residual scaffold is highlighted using arrows. Cell-free scaffolds had drifted away from the lesion site. Scale bars, 200 μm. **e** Locomotion recovery after SCI quantified by the Horizontal Ladder Walking Assay. Fault rates are presented as mean ± SEM (“uninjured control” and “scaffold + NSC” groups: *n* = 8 animals, “crush” and “scaffold-only” groups: *n* = 6 animals). The complete dataset is available in Supplementary Figs. 5b and 6). *P_1-way-ANOVA_ < 0.05, **P_1-way-ANOVA_ < 0.01. **f** Dot plot of locomotion fault rate at 10, 11, and 12 weeks post injury. Fault rates are presented as mean ± SD. *P < 0.05, **P < 0.01, ***P < 0.001 (Tukey’s post-hoc pairwise test assuming *P*_1-way-ANOVA_ < 0.05).

NSC-seeded porous collagen scaffolds were further evaluated in a mouse dorsal column crush model, a SCI model known to cause irreversible coordination dysfunction and hind limb instability^31^. The experimental design consisted of five animal groups (Fig. 4a, b). In the “uninjured control” group (*n* = 8 animals), following laminectomy, the dura matter was cut but the exposed spinal cord was not injured. In the “crush” group (*n* = 8 animals), the dorsal column of the exposed spinal cord was crushed using forceps. In the “scaffold + NSC” group (*n* = 10 animals), immediately following dorsal column crush, the lesion site was grafted with a porous collagen scaffold seeded with Bromodeoxyuridine (BrdU)-pulsed embryonic NSCs. In the “scaffold-only” control group (*n* = 8 animals), the lesion site was grafted with a cell-free porous collagen scaffold. Finally, in the “scaffold + NIH-3T3” control non-NSC group (*n* = 8 animals), the lesion site was grafted with a porous collagen scaffold seeded with mouse NIH-3T3 cells. NIH-3T3 fibroblast cells were chosen due to their proliferative capacity, end-term differentiation state (thus, as a non-NSC cell model), and robust fibroblast adhesion to PCS^25,28^.

**Fig. 5:**
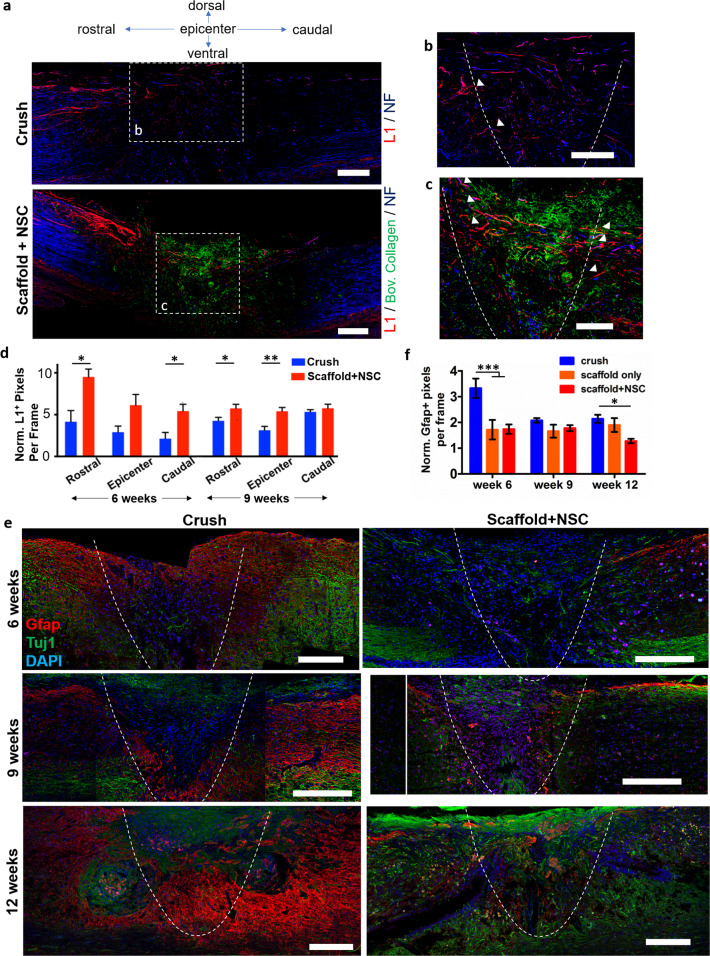
Porous collagen scaffolds seeded with embryonic NSCs increased axonal elongation and reduced astrogliosis at the lesion site after dorsal column crush. **a** Representative fluorescence images of parasagittal sections from crush and scaffold + NSC groups immunostained for neurofilament heavy chain, bovine collagen I and L1, 6 weeks post injury. Scale bars, 200 μm. **b**, **c** High-magnification images of regions shown in **a** highlight axons that stain for NF and L1 at the lesion (inside the graft) or on the approximate lesion boundary (dashed lines). Examples of L1^+^ axons crossing the approximate lesion boundary are marked by triangles. Scale bars, 100 μm. **d** Quantification of L1^+^ pixel density in crush and scaffold + NSC groups, caudally, in the epicenter, and rostrally to the lesion, at 6 and 9 weeks post injury. Results are normalized with respect to the uninjured control group and are presented as mean ± SEM. **e** Fluorescence imaging of parasagittal sections stained for Tuj1 and GFAP at the lesion 6, 9, and 12 weeks post injury. The approximate lesion boundary is shown using dashed lines. Bars: 200 μm. **f** Quantification of GFAP^+^ pixel density in the crush and scaffold + NSC groups at 6, 9, and 12 weeks post injury. Results are normalized with respect to the uninjured control group and are presented as mean ± SEM. “Uninjured control” and “scaffold-only” groups: *n* = 3. “Crush” and “scaffold + NSC” groups: *n* = 5. *P < 0.05, ***P < 0.001 (Bonferroni post-hoc pairwise test assuming *P*_1-way-ANOVA_ < 0.05).

**Fig. 6:**
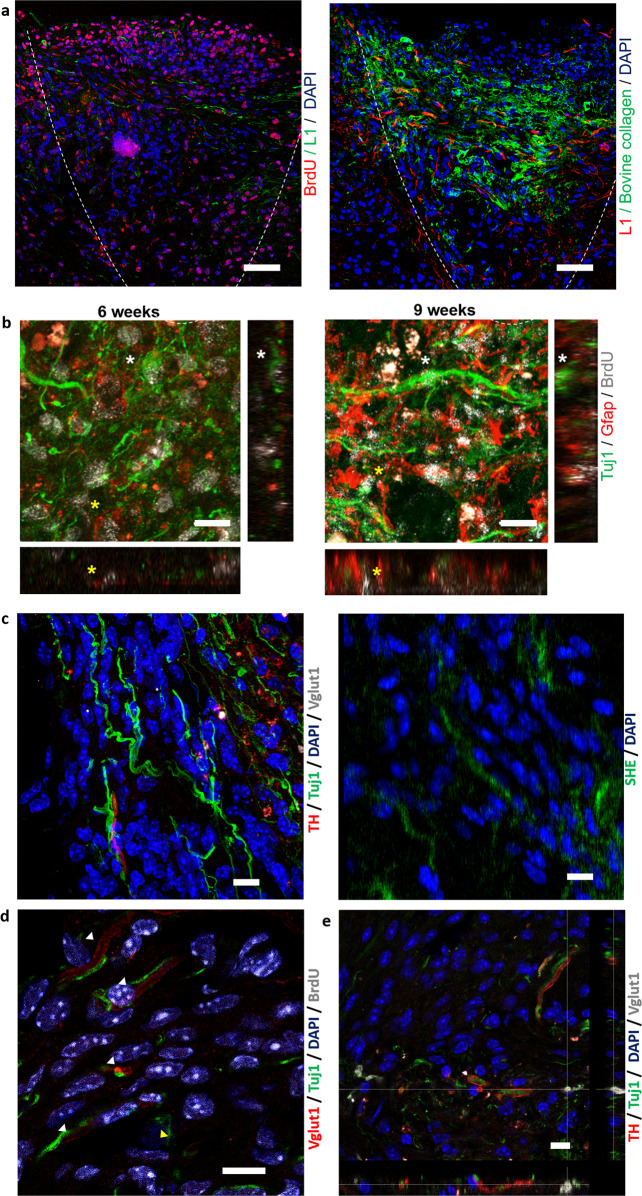
Porous collagen scaffolds safely delivered embryonic NSCs at the SCI lesion and enabled NSC differentiation towards both neurons and astrocytes in vivo. **a** Representative immunofluorescence images of sagittal sections stained for bovine collagen I, BrdU, and L1 at the lesion epicenter, 6 weeks post injury. Dashed line indicates the approximate lesion boundary. Scale bars, 50 μm. **b** High-magnification immunofluorescence images and orthogonal views at the lesion epicenter, 6 and 9 weeks post injury. Cellular colocalization of BrdU^+^ nuclei with cytoplasmic Tuj1 or GFAP are marked by white and yellow asterisks, respectively. Scale bar, 10 μm. **c** Immunofluorescence images of a parasagittal section acquired inside the graft 6 weeks post injury. Left: section stained for TH, Vglut1, and Tuj1. Right: second harmonic emission by residual scaffold collagen in the same location. Scale bar, 10 μm. **d**, **e** Immunofluorescence image of a parasagittal section acquired inside the graft 6 weeks post injury. The sections were stained for Vglut1, Tuj1, and BrdU (**d**) or Vglut1, Tuj1, and TH (**e**). Scale bars, 10 μm.

Histology revealed that NSC-seeded scaffolds integrated well with the surrounding tissue and no gaps were observed between the scaffold and the surrounding tissue 6, 9, and 12 weeks post injury (Figs. 4c, d, 5, and 6, and Supplementary Fig. 3). In the scaffold-only group, grafts had drifted away from the lesion site in four out of four animals examined 6 weeks post injury (Fig. 4c, d and Supplementary Fig. 3). In the scaffold + NIH-3T3 group, four out of eight animals showed signs of poor health and distress (weight loss, back feet paralysis, and heavy abdominal breathing) and were killed 2 weeks post injury. Post-mortem tissue histological evaluation of the killed animals showed extended damage and overt inflammatory response of the tissue surrounding the lesion (Supplementary Fig. 4a, b). Despite NSC proliferative capacity, such complications were not observed in the scaffold + NSC group. The remaining four animals of the scaffold + NIH-3T3 group were monitored for locomotion recovery and were used for histological analysis. Six weeks post injury, in surviving “scaffold + NIH-3T3” group animals scaffold drift was not observed, the scaffold was integrated with the surrounding tissue; however, there were extended areas of cavitation in the tissue surrounding the scaffold (Fig. 4c).

Following SCI, locomotion recovery was quantified by the horizontal ladder walking assay over a period of 12 weeks post injury (Fig. 4e, f, Supplementary Figs. 4 and 5, and Supplementary Video 2). Locomotion fault rate (percentage of misplaced steps) remained unchanged in the uninjured (laminectomy-only) control group (*μ* = 7.5%, *σ* = 3.2%) over the 12-week evaluation period. The response of the four SCI groups (crush, scaffold + NSC, scaffold-only, and scaffold + NIH-3T3) were not statistically different 1 day (*P*_1-way-ANOVA_ = 0.433, *F* = 0.955) and 1 week (*P*_1-way-ANOVA_ = 0.726, *F* = 0.44) post injury, indicating that SCI groups suffered from the same degree of injury. Overall, the vast majority of detected misplacements were classified as “correction” and mainly as “partial placement” (Supplementary Fig. 5b), indicative that dorsal column crush mainly affects fine motility. Severe foot misplacements were observed up to 2 weeks post injury in four SCI groups. Over a 12-week post-injury period, there was a significant overall effect of the treatment (*P*_Repeated Measures 2-way-ANOVA_ = 0.0002, *F*[2,238] = 8.822, 3.57% of variation). Some spontaneous locomotion restoration was observed in all four SCI groups (significant overall effect of time, *P*_Reapeted Measures 2-way-ANOVA_ < 0.0001, *F*[13,238] = 8.822, 44.38% of variation) in agreement with similar studies^32^. Starting at 3 weeks post injury, locomotion performance in the scaffold + NSC group was consistently better compared with the crush group. These differences became statistically significant at 10 (10.0 ± 1.8% vs. 19.2 ± 1.0%, *P*_Tukey_ < 0.05; *P*_1-way-ANOVA_ = 0.0072, *F* = 6.69), 11 (9.6 ± 0.9% vs. 16.9 ± 2.1%, *P*_Tukey_ < 0.05; *P*_1-way-ANOVA_ = 0.0031, *F* = 8.27) and 12 (8.4 ± 0.8% vs. 16.6 ± 1.6%, *P*_Tukey_ < 0.01; *P*_1-way-ANOVA_ = 0.0005, *F* = 12.33) weeks post injury (Fig. 4f). Scaffold + NSC was the only animal group where the slope *α* of the linear trendline that fits locomotion performance between 3 and 12 weeks post injury was negative and statistically non-zero (Scaffold + NSC: *α* = −0.79%/week; *P*_slope_ = 1.5 × 10^−5^. Scaffold-only: *α* = −0.014%/week; *P*_slope_ = 0.96. Crush: *α* = −0.18%/week; *P*_slope_ = 0.52. Uninjured control: *α* = −0.13%/week; *P*_slope_ = 0.34; Supplementary Fig. 6). Finally, scaffold + NSC was the only SCI group whose locomotion performance at 12 weeks post injury was not statistically different from one of the uninjured (laminectomy-only) control group (8.4 ± 0.8% vs 7.1 ± 0.8%, *P*_2-sided *t*-test_ > 0.2; Fig. 4f). Similar trends were observed in the response of the Foot Fault Score (Supplementary Fig. 5a), an alternative metric of locomotion performance that considers the type of foot misplacements. No improvement in locomotion recovery compared with the crush (injured, not grafted) group was observed in animals treated with porous scaffolds seeded with non-NSC NIH-3T3 cells (Supplementary Fig. 4c).

### NSC-seeded collagen scaffolds improved axonal elongation and reduced astrogliosis at the lesion site

Axon elongation through the lesion site is a key prerequisite for functional restoration after SCI. No significant axonal elongation was observed at SCI sites grafted with cell-free PCS grafts^16,19,23^. Six weeks post injury, NF^+^ axons were present caudally and rostrally of the lesion site. L1^+^ axons were present in the dorsal column adjacent to the lesion and in the lesion epicenter (Fig. 5a-d and Supplementary Fig. 7). At 6 weeks post injury, the scaffold + NSC group displayed significantly higher density of L1^+^ pixels compared with the crush group caudally (scaffold + NSC: 952 ± 4%, crush: 416 ± 134%, *n* = 5, *P* < 0.05) and rostrally (scaffold + NSC: 543 ± 82%, crush: 213 ± 74%, *n* = 5, *P* < 0.05; Fig. 5d). At 9 weeks post injury, the scaffold + NSC group displayed significantly higher density of L1^+^ pixels rostrally (scaffold + NSC: 580 ± 49%, crush: 489 ± 42%, *n* = 5, *P* < 0.05) and at the lesion epicenter (scaffold + NSC: 545 ± 45%, crush: 315 ± 47%, *n* = 5, *P* < 0.01). High-magnification images at the lesion epicenter 6 weeks post injury revealed significant numbers of NF^+^ axons inside the scaffold, many of which stain for L1, and multiple L1^+^ axons crossing the scaffold-tissue interface both rostrally and caudally (Fig. 5c).

Astrogliosis at the lesion site was evaluated by immunostaining for GFAP and calculating the fraction of GFAP^+^ pixels around the approximate lesion boundary. Six weeks post injury, intense GFAP staining was localized rostrally and caudally of the lesion epicenter in the crush group, a characteristic pattern of astrogliosis (Fig. 5e, f and Supplementary Fig. 8a, b). The fraction of GFAP^+^ pixels in the scaffold + NSC group was significantly smaller compared with the crush group at 6 weeks post injury (scaffold + NSC: 174 ± 18%, crush: 332 ± 37%, *n* = 5, *P* < 0.001) and at 12 (scaffold + NSC: 128 ± 9%, crush: 214 ± 16%, *n* = 3, *P* < 0.05) weeks post injury (Fig. 5f and Supplementary Fig. 8c), suggesting that the graft managed to reduce astrogliosis around the lesion. Microgliosis was also evaluated in the crush and scaffold + NSC groups by quantifying the fraction of pixels that stain for IBA1, a marker of microglia and recruited monocytes/macrophages. Images reveal that IBA1^+^ cells of phagocytotic morphology are attracted in the lesion site in both groups (Supplementary Fig. 9a). No statistically significant difference in the fraction of IBA1^+^ pixels was observed between the crush and scaffold + NSC groups (Supplementary Fig. 9b). Second harmonic emission (SHE) imaging revealed that although the graft did not manage to completely block the formation of a collagenous scar, in the scaffold + NSC group the collagen fibers of scar were localized mostly at the lesion boundary and not inside the scaffold (Supplementary Fig. 10).

### Neural differentiation of NSCs inside porous collagen scaffolds in vivo

To track the fate of NSCs delivered inside PCS at the SCI lesion site, NSC-seeded collagen scaffolds were pulsed with BrdU prior to their grafting (Fig. 4a, b). Immunostaining revealed that most BrdU^+^ nuclei were located inside the scaffold suggesting that PCS achieved localized delivery of NSCs at the lesion site (Fig. 6a). Manual counting in confocal *z*-stacks (stack volume 2.69 ± 0.38 × 10^5^ μm^3^) from five animals of the scaffold + NSC group 6 weeks post injury revealed 375.4 ± 74.5 nuclei per imaging stack corresponding to cell density 1.37 ± 0.15 × 10^6^ cells/μl. The majority of these nuclei (86.6 ± 1.5%) stained for BrdU, corresponding to a density of 1.19 ± 0.14 × 10^6^ BrdU^+^ cells/μl. Although it is not possible to calculate the survival rate of implanted NSCs (due to NSC proliferation and scaffold degradation), the high density of BrdU cells and the lack of cell debris inside the scaffold (Fig. 6c-e) suggest that the scaffold managed to protect NSCs from the harsh conditions of SCI. Furthermore, a few BrdU^+^ nuclei were observed within 200 μm from the scaffold boundary, suggesting that the porous nature of PCS enabled NSC migration and integration into the surrounding tissue in vivo. No ectopic NSC colonies were observed within 1 mm away from the lesion site.

To evaluate whether NSCs seeded inside PCS can differentiate in vivo in the SCI site, sections from the scaffold + NSC group were simultaneously stained for BrdU, Tuj1, and GFAP. Imaging and manual cell counting revealed that 6 weeks post injury, a significant fraction of BrdU^+^ cells stained positively for either Tuj1 (22.9 ± 2.4%, *n* = 5) or GFAP (6.0 ± 1.4%, *n* = 5; Fig. 6b). As NSCs at the day of implantation stained positively for nestin (Fig. 1f), our results provide evidence that NSCs differentiated towards both neuronal or glial fates inside PCS after graft implantation in vivo, a prerequisite for developing NSC-based relay treatments for SCI.

Finally, to probe the nature of the neurons and axons present at the lesion site 6 weeks post injury, parasagittal sections from the scaffold + NSC animal group were immunostained for Vglut1 (marker of excitatory glutamatergic neurons), TH (marker of dopaminergic neurons), and GAD65/67 (marker of inhibitory GABAergic neurons). Imaging revealed the presence of both Vglut1, TH, and GAD65/67 staining in the lesion epicenter (Fig. 6c-e and Supplementary Figs. 11 and 12). Superimposing confocal images with SHE images verifies that Vglut1, TH, and GAD65/67 signals were indeed located inside the collagen scaffold (Fig. 6c and Supplementary Fig. 11). Simultaneous labeling of Vglut1 and BrdU suggests that several NSC-derived neurons could be excited by glutamatergic neurons (Fig. 6d). Very few BrdU^+^ neurons expressed TH or GAD65/67 (Fig. 6e and Supplementary Fig. 12). Overall, these findings suggest the presence of both excitatory and inhibitory neurons inside the scaffold in the lesion epicenter 6 weeks post injury.

## Discussion

The development of effective clinical treatments for SCI is an important unmet medical need due to the severe consequences of SCI to patients. Existing clinical treatments (small molecules and surgical procedures) have limited efficacy and short-term effects. Several methods utilized clinically to replace injured organs (e.g., transplantation and allografts) cannot be applied in the injured spinal cord. Induced regeneration appears to be a promising, yet challenging alternative for providing SCI patients valuable functional and sustainable recovery. Indeed, over the past two decades, much research focused on developing novel regenerative medicine treatments for SCI. Among them, cell therapy promises to utilize the emerging understanding on stem cell biology to replace lost cells at SCI sites^7^. In particular, SCI grafts based of embryonic NSCs have demonstrated impressive effects on axonal elongation and synapse formation in rodent and primate SCI models^9–11^. However, significant development is required to safely deliver NSCs at SCI lesions and harvest their potential in clinical practice. Strong evidence, including the poor efficacy reported in the first clinical trial of human NSCs in SCI patients^13^, suggests that the supporting matrix is a key mediator of NSC effects^10^. Although several biomaterials have demonstrated ability to regulate NSC phenotypes in vitro^24^, little is known on the underlying mechanisms that could guide the development of effective SCI grafts. Furthermore, those biomaterials that have demonstrated positive effects to NSC in SCI grafts^9–11,21^ have not received regulatory approval before, adding another obstacle towards the clinical translation of NSC treatments. Meanwhile, the few biomaterials that have been translated in the clinical practice of induced organ regeneration, including porous collagen-based scaffolds (PCS), have not contributed to the development of effective SCI grafts. Despite the ability of PCS to induce regeneration in peripheral nerve injuries, cell-free PCS grafts have provided limited results in SCI^16,19^. Little is known about the ability of PCS to harvest the regenerative capability of potent NSCs in SCI. In this study, we demonstrate that PCS can safely deliver and regulate embryonic NSCs at SCI sites leading to significant locomotion recovery in a mouse SCI model. We focused on the two seminal PCS due to their established clinical application in regenerative medicine and their proven ability to modulate wound healing^22,27^. Finally, we utilized mouse embryonic cortical NSCs due to their proliferative capacity, trilineage differentiation, easy availability (ensures the reproducibility of our findings), and relatively easy isolation and expansion.

The first part of the study quantified the effects of the two seminal PCS on key phenotypes of NSCs (proliferation, viability, and neuronal differentiation) and NSC-derived neurons (axonal elongation) in vitro. Seeded NSCs adhered robustly on PCS struts either as single cells or formed neurosphere-like aggregates inside PCS pores (Fig. 1d-f). Collagen-GAG scaffolds enhanced NSC proliferation and survival compared with GAG-free collagen scaffolds (Fig. 2b, c), in agreement with previous in vitro and in-vivo reports^33,34^. We hypothesize that Ch6S can emulate the extracellular matrix of the NSC niche at the adult sub-ventricular zone or the embryonic telencephalon, where abundant GAGs and proteoglycans regulate NSC fate and the migration of newly born neurons^35^. The presence of Ch6S in PCS did not affect the fraction of NSCs that differentiated towards Tuj1^+^ neurons (Fig. 2d, e). However, GAG-free PCS enhanced the maturation of NSC-derived neurons compared with collagen-GAG scaffolds (evaluated by morphological assays; Fig. 2f-h), in agreement with the inhibitory role of chondroitin sulfate proteoglycans (CSPGs) in neuronal synapse maturation^36^. Neural maturation was further evaluated by quantifying the spontaneous activity of NSC-derived neurons inside PCS. Field recordings revealed that at 7 DIV the frequency of spontaneous events in bicuculline-treated neurospheres formed inside collagen scaffolds was significantly larger compared with neurospheres formed inside collagen-GAG scaffolds (Fig. 3a-c). Here, the inhibitory/excitatory state of GABA_A_ receptors was utilized as a developmental marker. Although in the adult CNS GABA_A_ receptors are inhibitory, in the early stages of development GABA_A_ receptors have excitatory effects^37^. Our finding that at 7 DIV spontaneous activity was increased in response to bicuculine in NSCs grown in collagen scaffolds but not in collagen-GAG scaffolds suggests that GABA_A_ receptors had already switched into becoming inhibitory in 3D-C but not in 3D-CG.

Fluorescence imaging of cytosolic Ca^+2^ in NSCs grown inside PCS revealed the presence of periodic Ca^+2^ events, whose duration and mean period was significantly smaller in collagen scaffolds compared with collagen-GAG scaffolds at 5 DIV (Fig. 3d-g), in agreement with the delayed neural differentiation observed in CG scaffolds via electrophysiology measurements. Although we did not identify whether cells participating in such oscillations were neurons or astrocytes, this results support the hypothesis that collagen scaffolds favor the functional maturation of NSC-derived neural cells and demonstrate neural activity in NSC-derived neural cells inside PCS in vitro. Finally, the ability of GAG-free PCS to support the functional maturation of neurons was further supported by the formation of functional networks of interacting (synaptophysin-positive) primary DRG neurons inside porous collagen scaffolds (Supplementary Fig. 2).

The second part of the study evaluated the ability of PCS grafts to safely deliver NSCs at SCI lesion sites and enhance locomotion recovery in a clinically relevant model of SCI. GAG-free porous collagen scaffolds were solely utilized due to their superior ability to support neuronal differentiation in vitro and also due to the reported adverse effects of CSPGs on axonal elongation^38^. Previous studies on PCS graft effects in SCI utilized transection models, which provide unambiguous conditions for quantifying axonal regeneration; however, they are much more severe than common SCI in human patients (usually contusions). Here, PCS grafts (cell-free or seeded with NSCs or non-NSCs (NIH-3T3 cells)) were evaluated in a mouse dorsal column crush SCI model (Fig. 4a, b), known to irreversibly affect both ascending and descending pathways (including the challenging corticospinal tract). The chosen SCI model provides a lesion volume where a graft can be implanted and therefore has been utilized previously to quantify graft effects^21,39^. Histology revealed that both types of cell-seeded grafts remained in the lesion site, whereas cell-free PCS drifted away from the lesion site (Fig. 4c, d and Supplementary Fig. 3). These results clearly suggest that seeded cells were critical for graft adhesion with the surrounding spinal cord tissue, a prerequisite for direct interactions between the scaffold and host neural cells. However, the type of cells seeded inside scaffold proved to be critical as NIH-3T3 cells, in sharp contrast to NSCs, did not induce any locomotion recovery and even induced complications and mice mortality (Supplementary Fig. 4).

Locomotion recovery in the crush, scaffold-only and scaffold + NIH-3T3 groups over a 12-week period after SCI was partial (locomotion performance did not reach the one of the uninjured control group). Grafting the SCI lesion site with cell-free scaffolds or NIH-3T3-seeded scaffolds did not induce any improvement compared with the crush (injured but not grafted) group, in agreement with previous studies (Fig. 4e and Supplementary Fig. 4b)^16,19^. On the other hand, starting at 3 weeks post injury, animals grafted with NSC-seeded scaffolds had consistently lower fault rate compared with the crush group (Fig. 4e). The ~8% absolute improvement in fault rate induced by the scaffold + NSC group (corresponding to a statistically non-zero slope of 0.79%/week; Supplementary Fig. 6) became statistically significant compared with the crush group at 10, 11, and 12 weeks post injury (Fig. 4f). Noticeably, at 12 weeks post injury, the locomotion fault rate of the scaffold + NSC group was not statistically different from the uninjured control group.

To gain insight on how NSC-seeded PCS grafts can improve locomotion recovery, we quantified graft effects on four key elementary processes of SCI wound healing: cell-scaffold interactions, NSC survival and differentiation, axonal elongation, and astrogliosis. A significant fraction of grafted scaffolds remained non-degraded at the lesion site at 6 weeks post injury, in agreement with the reported 3-week in-vivo degradation half-life of PCS^27^. In contrast to cell-free scaffolds, NSC-seeded scaffolds made firm contact with the surrounding tissue (Fig. 4c, d) and no void spaces were observed either inside the graft or at the graft–tissue interface. Extensive scaffold contact with the surrounding tissue (a pattern observed in PCS-induced regeneration in skin and peripheral nerves^25,28^) can facilitate interactions of host neurons with grafted cells or the scaffold itself.

A major finding of our study is that PCS grafts safely delivered NSCs at SCI lesion sites, where they supported NSC neuronal differentiation and functional integration with the surrounding tissue. Delivered NSCs were confined within the lesion site as evident by the high density of BrdU^+^ cells (1.19 ± 0.14 × 10^6^ BrdU^+^ cells/μl.) observed inside the non-degraded scaffold 6 weeks post injury (Fig. 6a). Several studies have reported safety concerns regarding the formation of ectopic colonies when NSC suspensions are delivered at lesion sites via a syringe^40^. No ectopic colonies were observed in this study within 1 mm from the lesion site, possibly because NSC delivery via PCS avoids applying high pressure to tiny suspension volumes and does not suffer from NSC suspension leak away from the lesion site (during grafting, NSCs adhere robustly to the surrounding PCS). Furthermore, several BrdU^+^ nuclei were observed within 200 μm away from the approximate lesion boundary. We hypothesize that the porous nature of PCS and the robust PCS-tissue contact discussed above, facilitates NSC migration into the surrounding tissue, which can greatly enhance the functional integration of NSC-derived cells with the surrounding tissue. A significant fraction of BrdU^+^ cells double-stained either for Tuj1 (22.9 ± 2.4%) or for GFAP (6.0 ± 1.4%), suggesting that seeded NSCs managed to differentiate towards both neurons and astrocytes in vivo (Fig. 6b) inside PCS in the harsh conditions of a SCI site. Immunolabeling for markers relevant to neurotransmitter expression revealed the presence of Vglut1^+^ synapses of excitatory glutamatergic neurons, dopaminergic neurons and inhibitory GABAergic neurons inside the scaffold (visualized by SHE; Fig. 6d-e and Supplementary Figs. 11 and 12). Verification of the nature of NSC-derived BrdU^+^ neurons was more challenging, as very few neurons triple stained for BrdU, Tuj1, and one of the markers utilized (Vglut1, TH, GAD65/67). Further research is required to trace the nature of the observed NSC-derived Tuj1^+^ cells and their functional integration with the surrounding spinal cord tissue.

In agreement with our in vitro observations, NSC-seeded PCS grafts proved to be a permissible substrate for axonal elongation in vivo. Animals treated with grafts had significantly more L1^+^ staining elongating axons at the lesion site compared with animals of the crush group at 6 and 9 weeks post injury (Fig. 5a-d). In the scaffold + NSC group, large numbers of L1^+^ axons were observed inside the graft, whereas several L1^+^ axons crossed the approximate lesion boundary (Fig. 5c). By facilitating axonal growth at the lesion site, NSC-seeded PCS can potentially enhance the functional integration of NSC-derived neurons with neurons of the surrounding spinal cord tissue. Further research is required to trace the origin of the observed L1^+^ axons and verify whether NSC-derived neurons inside PCS grafts formed synapses with axons originating from the surrounding tissue. Although collagen I is not a component of spinal cord extracellular matrix and is associated with scar, this study provides evidence that primary neurons, NSCs, and NSC-derived neuronal cells adhered well to PCS, and that neurons can grow long axons on scaffold struts both in vitro (Fig. 1d-f, 2d, f, and 3d, and Supplementary Fig. 2) and in vivo (Fig. 5a-c and 6b-e, and Supplementary Figs. 11 and 12), in agreement with previous reports^21,41^.

Finally, NSC-seeded PCS significantly reduced astrogliosis, a major barrier of axonal elongation. Significantly less GFAP staining was observed around the lesion boundary in the scaffold + NSC group compared with the crush animal group 6 weeks post injury (Fig. 5e, f), in agreement with reports of reduced astrogliosis in rat SCI models treated with cell-PCS^16^. Second harmonic imaging of collagen fibers revealed the presence of several collagen scar fibers at the lesion boundaries but few inside the remaining scaffold (Supplementary Fig. 10). Collagenous scar is a hallmark of failed regeneration in adult organs^28^ and a consequence of increased astrogliosis. Furthermore, IBA1 staining revealed that the scaffold did not manage to significantly decrease microgliosis; however, it also did not exacerbate the inflammatory response at the lesion (Supplementary Fig. 9b).

The major implication of our findings is to suggest that a new generation of NSC treatments for SCI can be developed based on PCS, similar to FDA-approved scaffolds already utilized in the clinical practice of induced regeneration in severe skin and peripheral nerve wounds. Compared with NSC grafts that utilize hydrogels^9–11,13,21^, SCI grafts based on PCS offer several advantages: first, PCS provide both effective spatial confinement of NSCs and easy migration of NSCs into the surrounding tissue. Second, PCS physicochemical parameters (pore structure, chemical composition, and cross-linking) can be easily tuned to better modulate specific SCI pathophysiological processes (including astrogliosis and wound contraction^22,28^), enhance axonal elongation in challenging tracts, and improve NSC differentiation and functional integration^23^. Emerging bioengineering technologies (e.g., 3D printing, microfabrication, and chemical functionalization) can further customize graft structure to better match the structure of the surrounding neural tissue or adapt to the needs of individual patients^23,42–44^. Third, the long in-vivo degradation half-life of PCS enables elegant manipulation of seeded NSCs prior to graft implantation. Such manipulations can use emerging protocols for directing NSCs towards specific lineages^45^ in order to enhance graft effects on specific spinal cord tracts^11,44^. Finally, PCS-based SCI grafts could greatly benefit the clinical translation of emerging NSC technologies for SCI (derivation of safe patient-specific NSCs and optimization of NSC manipulations) by exploiting the extensive regulatory and clinical experience of existing FDA-approved PCS grafts.

## Methods

### Scaffold fabrication

Porous collagen and collagen-GAG scaffold sheets were fabricated as described previously^26^, by lyophilizing a 5 mg/ml microfibrillar collagen I suspension or a 5 mg/ml microfibrillar collagen I suspension supplemented with 0.44 mg/ml Ch6S, respectively. The resulting 2.5 mm-thick dry sheets were cross-linked via dehydro-thermal treatment (105 °C, 50 mTorr, 24 h). Scaffold structure was verified by SEM. Cylindrical scaffold samples (3 mm diameter) were cut using a biopsy punch.

### Primary neural cell isolation and culture

Animal experimentation protocols 27300 and 262272 were approved by the Veterinary Directorate of the Region of Crete and FORTH ethics committee. Experiments were carried out in compliance with EU guidelines 2010/63/EU. Reagents were obtained from Thermo Fisher Scientific unless otherwise noted. C57/BL6 mice were maintained on a 12 h light/dark cycle with ad libitum access to food and water.

For NSC isolation, pregnant mice (gestational day 13.5) were killed via cervical dislocation, embryos were carefully removed and washed gently in HBSS + P/S [Hanks’ balanced salt solution (HBSS), 5% penicillin/streptomycin], and NSCs were isolated by mechanically dissociating cortical hemispheres in NSC complete medium [Dulbecco’s modified Eagle’s medium (DMEM)/F12 (Sigma), N2 supplement, 2 mM l-glutamine, 0.6% d-glucose, 100 μg/ml primocin (InvivoGen), 20 ng/ml FGF2 (R&D), 20 ng/ml EGF (R&D)]. NSCs (2.5 × 10^5^) in 5 ml complete medium were seeded in T25 flasks. One milliliter of fresh medium was added every other day. Neurospheres formed within 2 days. Neurospheres were dissociated by accutase (Sigma) at day 4 or 5. Dissociated NSCs were either passaged or used for experiments (passage 3–8).

For DRG cell isolation, ~200 ganglia from 8 embryos settled for 5 min in HBSS + P/S medium on ice inside sterile 1.5 ml tubes, were incubated in 0.25% trypsin at 37 °C for 5 min prior to dissociation into single neurons by pipetting using a fire-polished Pasteur pipette in DRG medium (RPMI, 10% fetal bovine serum (FBS), 1% P/S, 100 ng/ml NGF (Millipore)). Cells from three dissociated ganglia were seeded in 3 mm-diameter scaffold samples. DRG medium was changed every other day until day 7.

### Cell culture inside PCS or on coverslips

Three-dimensional NSC culture utilized PCS samples (3 mm diameter and 2.5 mm height) maintained as floating sponges in 48-well plates. The day before cell seeding, wells were coated by air-drying 0.1% agarose overnight inside a sterile hood. NSCs were seeded in each scaffold by first placing the scaffold on agarose-coated wells, pipetting a 10 μl drop containing 3 × 10^4^ NSCs on the scaffold, incubating at 37 °C for 10 min, and finally adding 300 μl NSC complete medium. Two-dimensional NSC culture took place by plating 2.5 × 10^4^ NSCs on sterile coverslips coated with PDL and laminin (Sigma). In survival and proliferation experiments, NSCs were grown in complete medium. NSCs were fixed for immunocytochemistry at 3, 5, 7, and 10 DIV. In differentiation experiments, NSCs were grown in complete medium for 3 days and then in Neurocult differentiation medium (Stem Cell Technologies) for 4 more days, fixed, stained, and imaged. For each experimental treatment, at least three scaffold samples were quantified.

### Immunocytochemistry, confocal microscopy, and cytometry

At indicated days, NSCs grown on coverslips or inside porous scaffolds were washed twice in phosphate-buffered saline (PBS) and fixed by 4% paraformaldehyde (PFA) in PBS for 15 min at 4 °C. Samples were washed in PBS, blocked in 5% bovine serum albumin (BSA) in PBST (0.3% Triton X-100 in PBS) at room temperature for 1 h, incubated in primary antibodies (Ki67: 1:500 Abcam ab16667; β3 tubulin: 1:1000 Biolegend MMS-453P (Tuj1 clone); Olig2: 1:500 Novus NBP1-28667; GFAP: 1:1000 Millipore AB5541; Neurofilament heavy chain: 1:1000 Abcam ab4680; Synaptophysin: 1:500 Abcam ab7837; and Nestin: 1:1000 Novus NB100-1604) diluted in PBST overnight at 4 °C, washed twice in PBS, incubated in fluorophore-conjugated secondary antibodies (Thermo) diluted 1:1000 in PBST for 1 h at room temperature, washed twice in PBS, and counterstained with 4′,6-diamidino-2-phenylindole (DAPI). In viability experiments, samples were also counterstained with 1 μM propidium iodide.

Fluorescently labeled samples were imaged in a Leica TCS SP8 inverted confocal microscope using a ×20 oil-immersion objective lens (Leica Microsystems, Wetzlar, Germany). Three *z*-stacks per sample (1 μm spacing) were acquired. Images were processed by custom MATLAB software (Mathworks, Natick MA; available upon request) that consisted of three steps as follows: (1) identification of cell nuclei at each plane by h-min morphological filtering on the DAPI channel, followed by user inspection and correction. (2) Identification of unique cell nuclei over the complete *z*-stack based on the localization of nuclei centroids and nuclei size statistics. (3) Single-cell calculation of markers of interest (Ki67 and propidium iodide). Threshold levels, chosen manually by two expert users, were applied to all images. In differentiation experiments, GFAP^+^, Tuj1^+^, and Olig2^+^ cells were counted manually based on confocal *z*-stacks due to staining pattern complexity. For the assessment of neurite branching, the number of dendrites emerging from the main neuronal axon were counted manually. Neuron axon length was calculated by tracing 30 axons stack-by-stack using Fiji software. For the assessment of BrdU/Tuj1/GFAP double labeling, three confocal stacks (~129 × 129 × 6 μm each) per animal (*n* = 5 animals) were acquired within the scaffold. Cell nuclei were counted based on distinct DAPI objects. Cell density (cells/mm^3^) was calculated based on the volume of the acquired *z*-stack. Tuj1^+^/BrdU^+^ neurons or GFAP^+^/BrdU^+^ astrocytes were counted manually based on BrdU^+^/DAPI^+^ nuclei surrounded by Tuj1 or GFAP staining.

### Scanning electron microscopy

All steps took place at 4 °C. Cell-seeded scaffolds were washed twice in 0.1 M sodium cacodylate buffer (SCB) for 15 min, fixed in 2% glutaraldehyde, 2% formaldehyde in 1% SCB for 1 h, washed twice in 1% SCB for 15 min, and dehydrated in serial ethanol solutions. Samples were sputter-coated by a 10 nm-thick gold layer (Humme Technics, Inc., Alexandria, VA, USA) and imaged in a JEOL 7000 scanning electron microscope (JEOL, Tokyo, Japan) at 15 kV voltage.

### Electrophysiology

Field recordings were performed on NSC neurospheres grown in 2D, 3D-C, and 3D-CG cultures at 3, 5, and 7 DIV. Artificial cerebrospinal fluid (125 mM NaCl, 7.5 mM KCl, 26 mM NaHCO_3_, 3 mM CaCl_2_, 10 mM glucose pH 7.4, 315 mOsm/l) oxygenated with 95% O_2_ was continuously perfused during recordings. An extracellular recording electrode filled with 2 M NaCl was placed inside neurospheres identified via DIC bright-field microscopy using an Axioskop 2FS microscope (Carl Zeiss, Inc., Jena, Germany). Voltage responses were amplified by a BVC-700A amplifier (Dagan Corp., Minneapolis, MN, USA) and digitized by a ITC-18 board (Instrutech, Inc.) on a PC. Data were acquired and analyzed using custom scripts in IgorPro software (Wavemetrics, Inc., Lake Oswego, OR, USA). Spontaneous field excitatory postsynaptic potential traces were acquired for 20 min before the addition of bicuculine (Tocris) and for 30 min after. Spontaneous events were identified as voltage responses larger than four times the SD *σ*_b_ of the background signal. The average number of spontaneous events for each sample was calculated from traces covering the last 5 min before the addition of bicuculine and the last 5 min of the 30 min recording period after the addition of bicuculline.

### Calcium imaging

NSCs were seeded in collagen or collagen-GAG scaffold samples and grown for 5 or 7 days in NSC complete medium. Samples were stained by 4 μM Fluo3 in NSC complete medium for 45 min, washed twice, placed in complete medium supplemented with 7.5 mM KCl on a glass-bottom petri dish, and imaged in a SP8 confocal microscope or in a custom-built multiphoton microscope based on an Axio Observer Z1 (Carl Zeiss, Inc.) employing a Pharos-SP fs oscillator (Light Conversion, Vilnius, Lithuania) at 1030 nm, 70–90 fs, 76 MHz. Confocal images were acquired by a ×40/1.2NA water-immersion objective (300 μs pixel dwell time, 2 fps, 256 × 256 pixels). Multiphoton images were acquired by a 20×/0.8NA objective (320 μs pixel dwell time, 3 fps, 250 × 250 pixels). Videos were processed by custom MATLAB software. Regions of interest (ROIs) corresponding to cells where Ca^+2^ oscillations took place were identified manually. For each ROI, the time profile of Fluo3 emission was analyzed in order to measure event duration (FWHM) and mean inter-event periodicity.

### Spinal cord injury model

The week prior to injury, mice (male C57/BL6, 1.5–2 months old) settled down in the animal facility and walked through the ladder to become familiarized. Three days prior to injury, 3 × 10^4^ cells (NSCs in the “scaffold-NSC” group and NIH-3T3 in the “scaffold-NIH-3T3” group) were seeded into 1 × 1 × 1.5 mm collagen scaffold samples in medium (complete NSC medium for NSCs, DMEM supplemented with FBS, and pen/strep for NIH-3T3). Starting 2 days prior to injury and repeating once every 12 h, cell-seeded scaffolds were pulsed with 10 nM BrdU. Prior to implantation, grafts were washed for 30 min in PBS.

All surfaces, tools, and instruments utilized in the following procedure had been sterilized carefully. Before anesthesia, a drop of metacam was introduced per os. Mice were anesthetized by 2.5:1 isoflurane:oxygen mix inhalation for 5 min in a scavenger box until breathing slowed down. After verifying the absence of paw reflexes, each mouse was shaved in the level of the humpback and the exposed skin was disinfected with Hibiscrub. Each mouse was then transferred on a heat-pad, ophthalmic ointment was applied to avoid eye dryness and 2:1 isoflurane:oxygen mix was applied to maintain deep anesthesia via a mask. A skin incision was made from the base of the humpback until the higher point of the rib cage, and exposed muscles were carefully torn. After removing surrounding ligaments, the bone of T10 vertebra was removed and an incision was made in the dura matter above the T13 segment. The dorsal column crush was applied by inserting Dumont #5 fine forceps 1 mm deep into the white matter and keeping them closed for 10 sec. This step was repeated once, to create an ~1 mm^3^ pocket (lesion). In the crush animal group, the lesion was not grafted. In the scaffold-only group, a cell-free collagen scaffold was placed in the pocket. In scaffold + NSC and scaffold + NIH-3T3 groups, a cell-seeded collagen scaffold was placed in the pocket. In all animal groups, the exposed spinal cord was covered by a hemostatic sponge (Geistlich Bio-Gide) to protect the exposed spinal cord tissue, the muscles and skin were sutured, the skin was disinfected, and metacam was provided for the next 3 days.

### Functional evaluation of locomotion recovery via the ladder walking assay

Mice walked along a horizontal ladder consisting of two parallel 100 × 25 cm acrylic sheets connected by ø2 mm aluminum rods spaced ~1 cm. The ladder was elevated at cage height above ground. Animals were trained to cross the ladder from a neutral cage to their home cage in the same direction. Before each session, mice crossed the ladder twice for habituation. Each animal run the assay five times per session. Videos of walking mice were analyzed in frame-by-frame by two researchers blind to the condition (group, week) of the animal analyzed to identify locomotion errors, defined as steps where a hind limb missed or slipped off or was misplaced on a rod. Each fault was classified using a 7-category scale (0: total miss, 1: deep slip, 2: slight sleep, 3: replacement, 4: correction, 5: partial placement, 6: correct placement) as described previously^46^. Locomotion performance was described via the fault rate (fraction of steps classified in categories 0–5) or via the Foot Fault Score^46^. The last step before and the first step after a stop were excluded; only consecutive steps were included in the analysis. Only animals where statistically significant (fault step rate > 15%) locomotion deterioration was observed 1 day or 1 week post injury were considered for further ladder assay analysis and histological evaluation.

### Histological evaluation of recovery after SCI

Six, 9, or 12 weeks post SCI, mice were deeply anesthetized using 2.5:1 isoflurane:oxygen mix and transcardially perfused with ice-cold heparinized (10 U/mL) saline followed by 4% PFA in PBS. Spinal cords were dissected, post-fixed in 4% PFA at 4 °C for 1 h, washed in PBS, and immersed in 30% sucrose solution in 0.1 M PB for 24 h at 4 °C. The 2 mm-long part of the spinal cord tissue centered around the lesion was frozen in isopentane at −70 °C and 20 μm-thick parasagittal sections were cut in superfrost slides.

For histological analysis, slides were placed in ice-cold acetone for 5 min, air-dried for 10 min in laminar flow, washed twice in PBS, fixed for 15 min in 4% PFA in PBS at 4 °C, washed in PBS, blocked in PBST (0.3% Triton X-100 in PBS) supplemented with 5% BSA at room temperature for 1 h, and incubated in primary antibodies (L1: 1:000 rabbit polyclonal antibody; GFAP: 1:1000 Millipore AB5541; BrdU: 1:200 Abcam ab6326; β3 tubulin: 1:1000 Biolegend MMS-453P (Tuj1 clone); Neurofilament heavy chain: 1:1000 Abcam ab4680; IBA1: 1:1000 Wako 019-19741; Vglut1: 1:400 Synaptic systems 135304; Tyrosine Hydroxylase: 1:1000 Abcam ab6211; GAD65/67: 1:1000 Abcam ab11070-50; and Collagen I: 1:100 Novus Biologicals NB600-1408) diluted in PBST overnight at 4 °C, washed twice with PBS, incubated with fluorophore-conjugated secondary antibodies (Thermo) diluted 1:1000 in PBST for 1 h at room temperature, washed twice in PBS, mounted, and counterstained with DAPI. For BrdU^+^ quantification, sections were incubated in 2 N HCl at 37 °C for 30 min, rinsed in 0.1 M sodium tetraborate pH 8.5 for 10 min, and rinsed twice in PBS before blocking. Stained sections were imaged in a Leica TCS SP8 inverted confocal microscope.

Immunostained parasagittal sections were imaged at the epicenter, caudally and rostrally to the lesion. Positive staining (L1, GFAP, or IBA1) was defined by thresholding using a common value chosen by an expert user. Measurements from four randomly selected fields were averaged per section. Results from six sections were averaged per animal. Axonal elongation was quantified by calculating the percentage of pixels that stained positively for L1 in the dorsal column in the immediate proximity (caudally and rostrally) of the lesion boundary and in the lesion epicenter. Care was taken to avoid the background L1 staining observed in the spared tissue, mostly in the gray matter (Supplementary Fig. 7). Astrogliosis was quantified by calculating the percentage of pixels that stained positively for GFAP immediately rostrally and caudally to the lesion boundary. Microgliosis was quantified by calculating the percentage of pixels that stained positively for IBA1 in the lesion epicenter. All pixel measurements were normalized to the mean measurement of the uninjured control animal group.

For visualization, acquired images were processed as follows: (1) for each channel (corresponding to a specific fluorophore or SHE), the mean of the background signal (calculated in a region where no signal is expected) was subtracted from the signal intensity. (2) Channel intensity was then scaled linearly with respect to a common intensity value (chosen usually as 90–98% of peak intensity).

### Statistical analysis

Experimental data are expressed as mean ± SEM. Statistical analysis was performed using the Prism software (Graphpad, La Jolla, CA, USA). Statistical significance was assessed by unpaired two-tailed Student’s *t*-tests (two-group comparisons) or analysis of variance (ANOVA) followed by Tukey’s or Bonferroni’s post-hoc test (multi-group comparisons) assuming a statistical significance level of 0.05. To minimize type II error, statistical comparison of locomotion performance against the uninjured control group was assessed by unpaired two-tailed Student’s t-tests assuming a significance level of 0.1. For in-vivo experiments, the sample size required was estimated based on the number of groups and the expected effect size using the G^*^power software (Düsseldorf, Germany). Due to the lack of published data on experimental variability in dorsal column crush injuries treated with biomaterial grafts, initially power was chosen at 80%.

### Reporting summary

Further information on research design is available in the Nature Research Reporting Summary linked to this article.

## Supplementary information

reporting summary

supplementary material

supplemental movie s1

supplemental movie s2

## Data Availability

All relevant data supporting the findings of this study are available within the paper, its supplementary information and from the corresponding authors upon reasonable request.
