# Supplementary material for: Neural stem cell delivery via porous collagen scaffolds promotes neuronal differentiation and locomotion recovery in spinal cord injury
Source: NPJ Regen Med. 2020 Jun 15;5:12. doi: 10.1038/s41536-020-0097-0 (PMC7295991; doi:10.1038/s41536-020-0097-0)
Supplement: Supplementary file 2 — supplementary material [file 41536_2020_97_MOESM2_ESM.pdf]

SUPPLEMENTARY MATERIALS

Supplemental Videos

Supplementary Video 1. Representative movie of Ca^{+2} oscillations in NSC seeded inside a porous collagen (GAG-free) scaffold, 5 DIV.

Supplementary Video 2. Representative horizontal ladder walking assay movie clips 1 day before injury (“uninjured control” animal group), 1 day after injury (“crush” and “scaffold+NSC” animal groups) and 12 weeks post injury (“uninjured control”, “crush”, “scaffold+NSC”, “scaffold only” and “scaffold+NIH3T3” animal groups).

Supplemental Figures

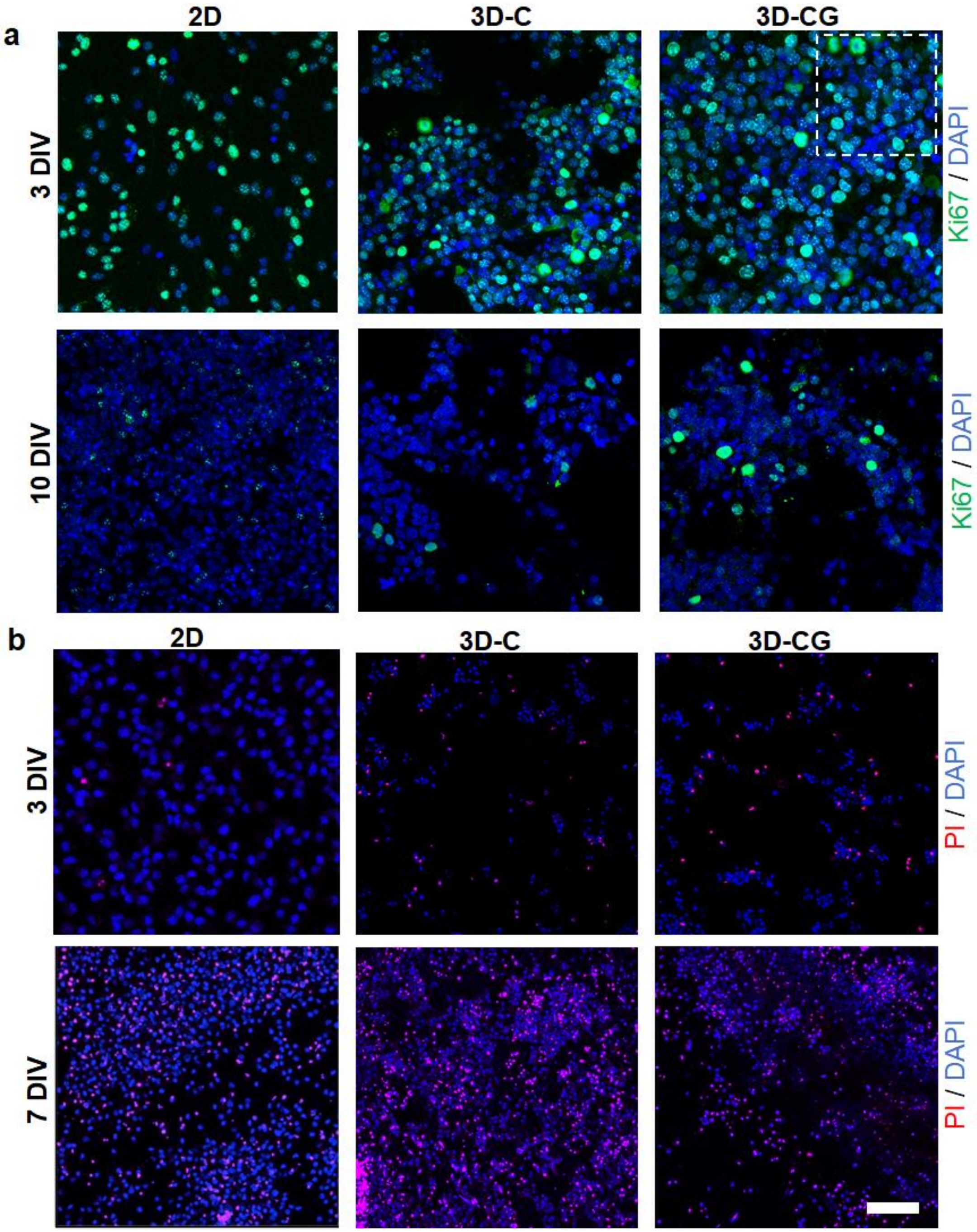

Supplementary Figure 1. The effect of PCS composition on NSC proliferation and viability *in vitro*. (**a**) Representative confocal images of NSCs immunostained for Ki67 and DAPI in 2D, 3D-C and 3D-CG cultures at 3 and 10 DIV. The white box corresponds to the area shown in Fig. 2a. (**b**) Representative confocal images of NSCs in 2D, 3D-C and 3D-CG cultures stained by propidium iodide and DAPI at 3 and 7 DIV. Scale bars, 50 μm.

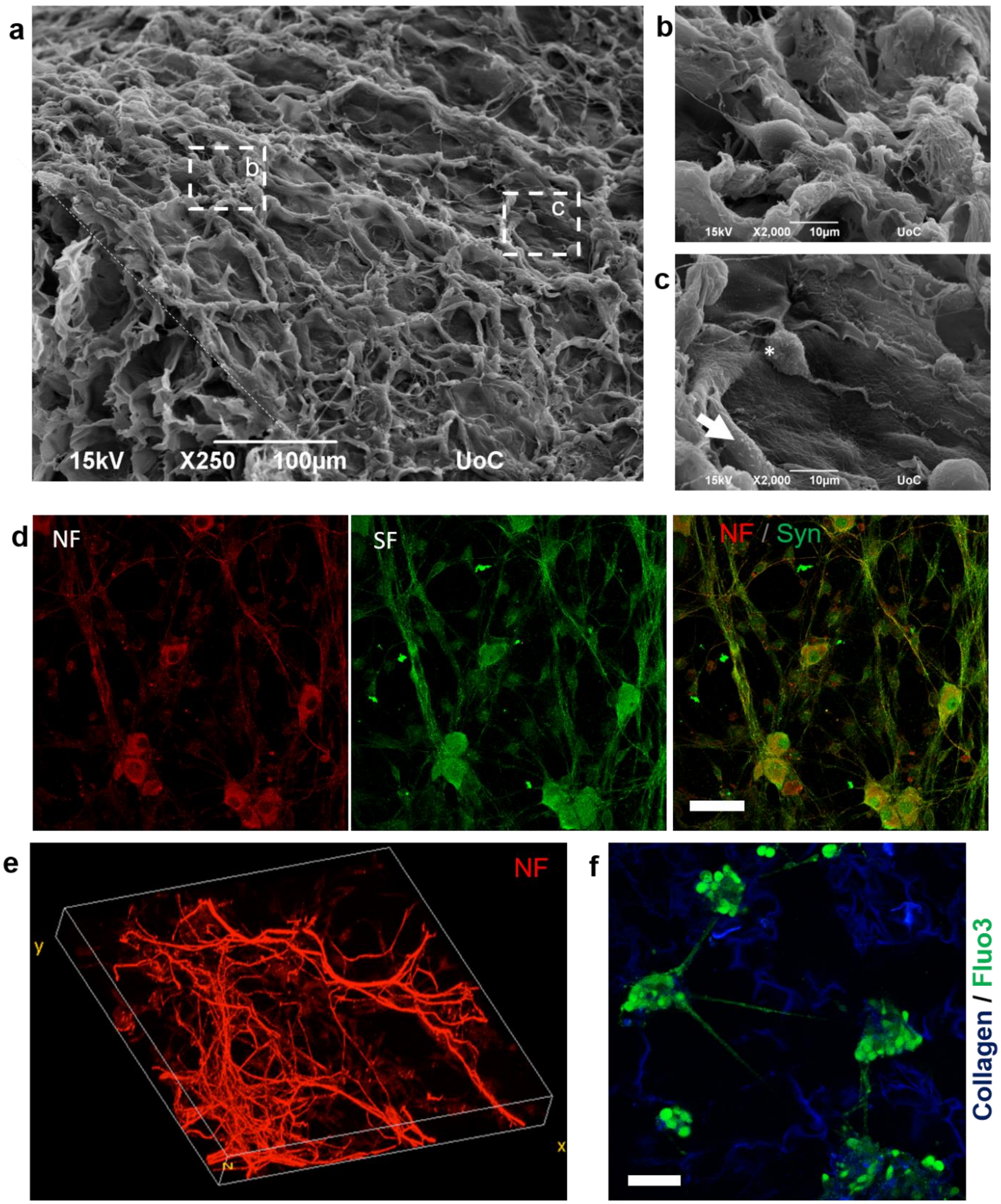

Supplementary Figure 2. 3D culture of CNS and PNS neural cells inside porous collagen scaffolds. (**a**) SEM image of DRG cells inside a porous collagen scaffold (3D-C) at 7 DIV. Dashed line highlights the interface between the scaffold surface (right side) and the scaffold interior (left side). (**b,c**) High-magnification images of regions shown in A demonstrate robust DRG cell attachment on the scaffold,

including neurons (asterisk) and Schwann cells (arrow). (**d**) Immunofluorescence imaging of synaptophysin and neurofilament expression in DRG cells grown inside a porous collagen scaffold. Scale bar, 50 μm. (**e**) 3D reconstruction of a network of DRG neurons (immunostained for neurofilament heavy chain) formed inside a porous collagen scaffold at 7 DIV. Box size: 263×263×80 μm. (**f**) Immunofluorescence image of live fluo3-stained spinal cord cells (motor neurons and interneurons derived from mouse embryonic stem cells) seeded and grown inside a collagen scaffold for 5 days. Scale bar, 50 μm.

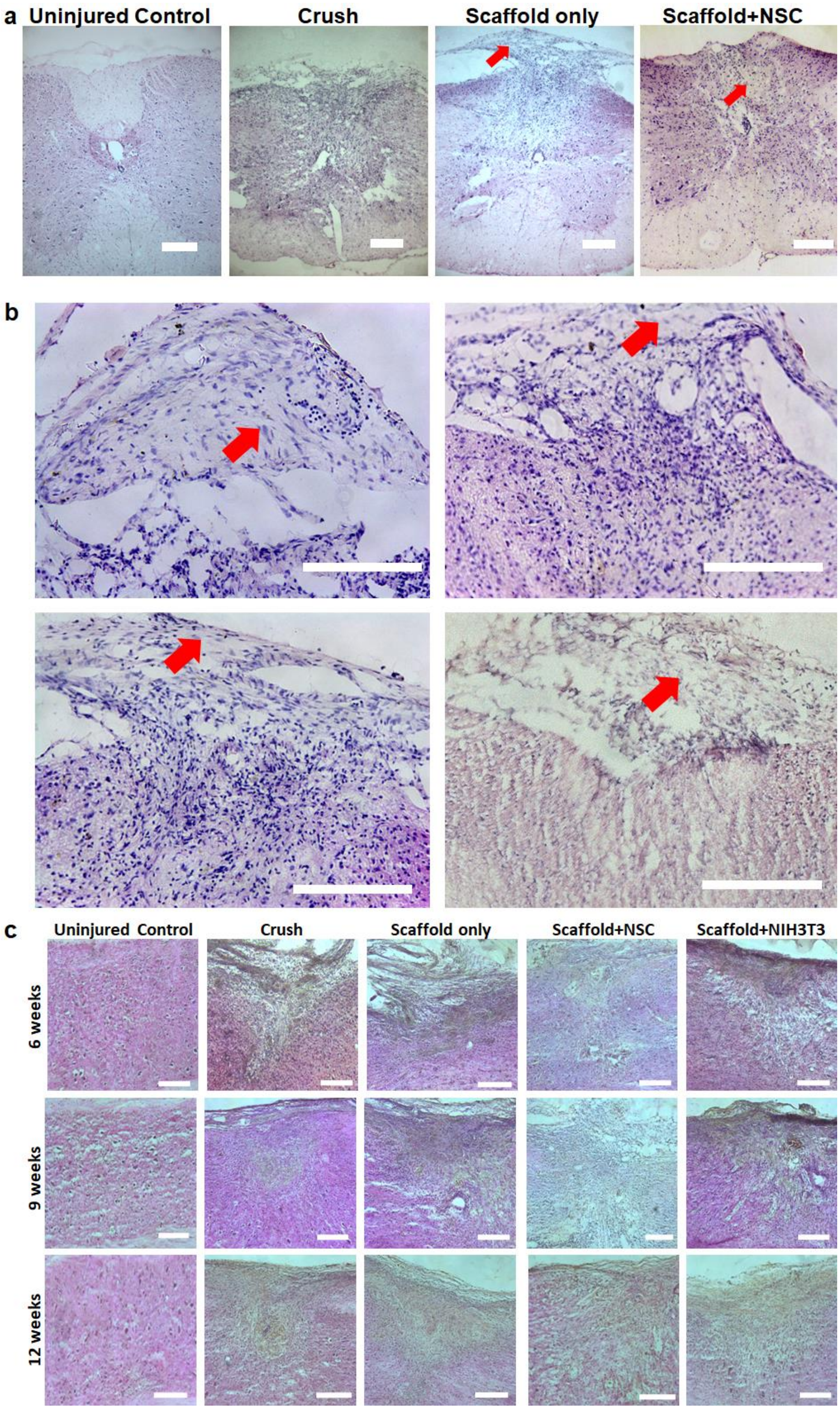
a
Uninjured Control
Crush
Scaffold only
Scaffold+NSC
b
c
Uninjured Control
Crush
Scaffold only
Scaffold+NSC
Scaffold+NIH3T3
6 weeks
9 weeks
12 weeks

Supplementary Figure 3. Cell-free porous collagen scaffolds drifted away from the SCI site while cell-seeded porous collagen scaffolds remained in the lesion site. (**a**) H&E staining of spinal cord cross sections from uninjured control (laminectomy only), crush (no graft), scaffold only (cell-free collagen scaffold graft) and scaffold+NSC animal groups 6 weeks post-injury. (**b**) Higher magnification images of spinal cord cross sections stained with H&E from four animals grafted with cell-free collagen scaffolds, 6 weeks post-injury. Arrows indicate the location of residual scaffold. Scale bars, 200 μm. (**c**) H&E staining of spinal cord parasagittal sections from all animal groups 6, 9 and 12 weeks post-injury. Scale bars, 200 μm.

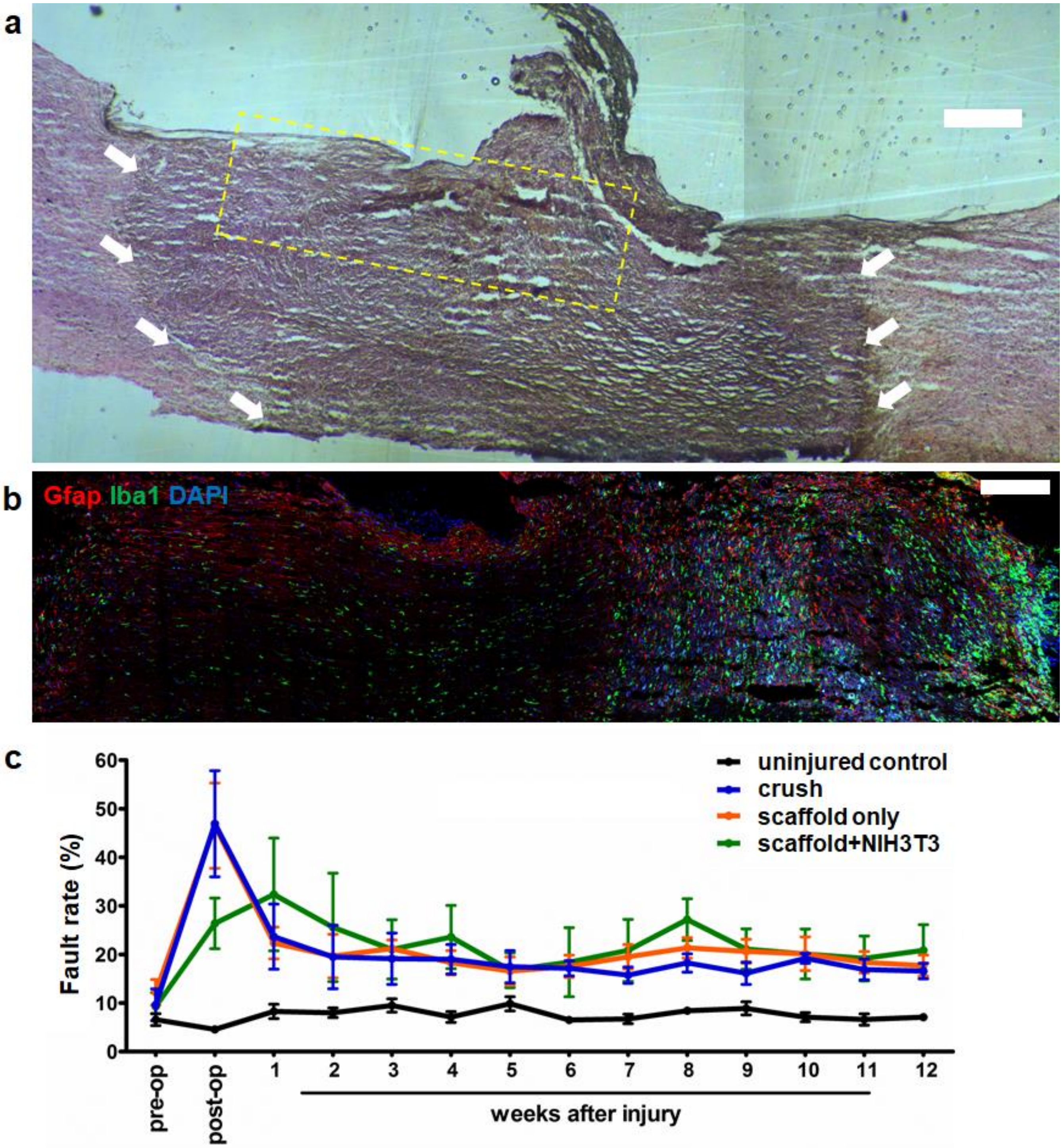

Supplementary Figure 4. No improvement in locomotion recovery was observed in injured mice grafted with porous collagen scaffolds seeded with NIH-3T3 cells instead of neural stem cells. (**a**) Image of a spinal cord parasagittal section stained with H&E from a mouse grafted with NIH-3T3-seeded porous collagen scaffold. The mouse was euthanized 2 weeks post-injury due to distress. Arrows highlight that a

significant part of the spinal cord tissue was severely damaged. Scale bar, 400 μm. (**b**) Representative fluorescence images of the same region as (a) highlighting severe astrogliosis ($GFAP^+$ cells) and microgliosis ($IBA1^+$ cells). Scale bar, 200 μm. (**c**) Locomotion recovery after SCI quantified by the Horizontal Ladder Walking Assay in the uninjured control, crush and scaffold+NIH3T3 animal groups. Fault rates are presented as mean ± s.e.m. (uninjured control: n = 8 animals, crush: n = 6 animals, scaffold+NIH3T3: n = 4 animals). Animals euthanized were not included in this plot). At no time was the locomotion performance of the scaffold+NIH-3T3 group statistically different from the performance of the crush group.

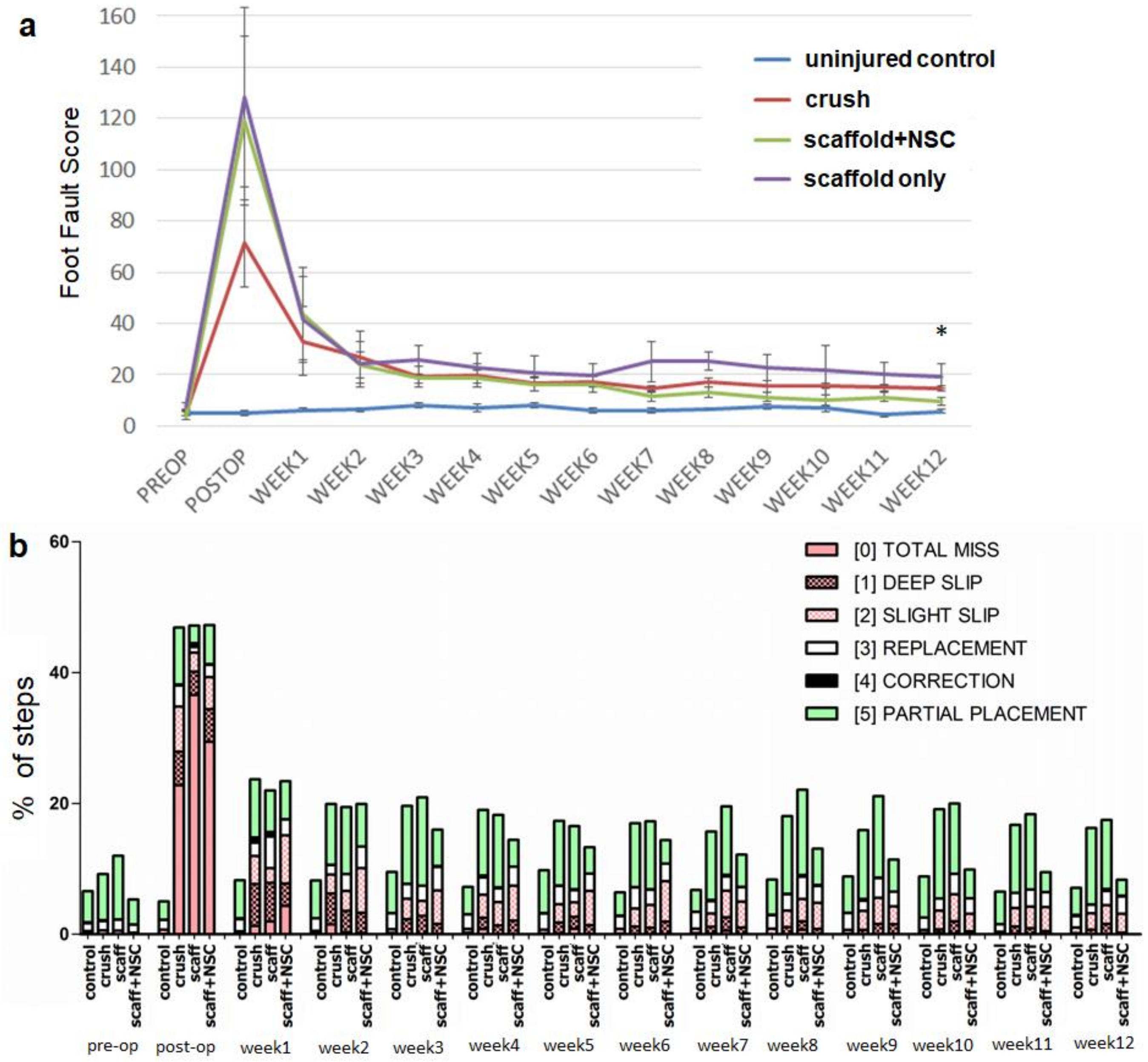

Supplementary Figure 5. Further analysis of horizontal ladder walking assay dataset. (**a**) Foot Fault Score response per group over the 12-week recovery period after SCI. Scores are presented as mean ± s.e.m. $^{*}P_{\text{1-way ANOVA}}<0.05$. (**b**) Classification of misplaced steps according to the procedure described previously[46] per group per time point.

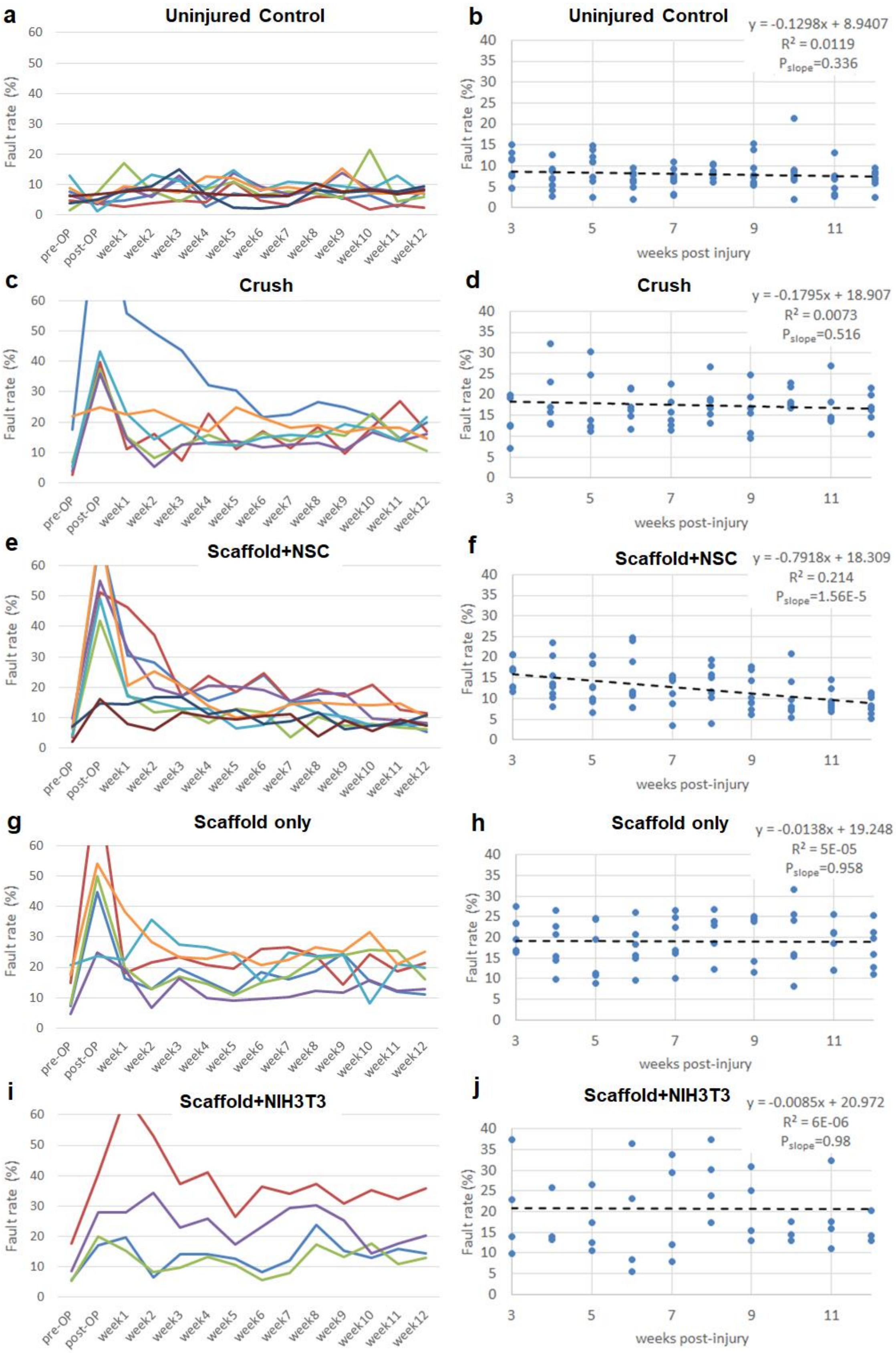
a
Uninjured Control
Fault rate (%)
pre-OP
post-OP
week1
week2
week3
week4
week5
week6
week7
week8
week9
week10
week11
week12
b
Uninjured Control
y = -0.1298x + 8.9407
R² = 0.0119
P_slope=0.336
weeks post injury
c
Crush
d
Crush
y = -0.1795x + 18.907
R² = 0.0073
P_slope=0.516
weeks post-injury
e
Scaffold+NSC
f
Scaffold+NSC
y = -0.7918x + 18.309
R² = 0.214
P_slope=1.56E-5
g
Scaffold only
h
Scaffold only
y = -0.0138x + 19.248
R² = 5E-05
P_slope=0.958
i
Scaffold+NIH3T3
j
Scaffold+NIH3T3
y = -0.0085x + 20.972
R² = 6E-06
P_slope=0.98

Supplementary Figure 6. Raw data of the horizontal ladder walking assay utilized to evaluate locomotion recovery over a 12-week period after dorsal column crush SCI. (**a,c,e,g,i**) Fault rate response per animal per group. Each curve corresponds to a different animal. (**b,d,f,h,j**) Linear trendlines ($y = ax + \beta$) that fit the fault rate response per group between 3 and 12 weeks post injury, along with the corresponding R^2 statistic and the p-value P_{slope} for the statistical test of non-zero slope a.

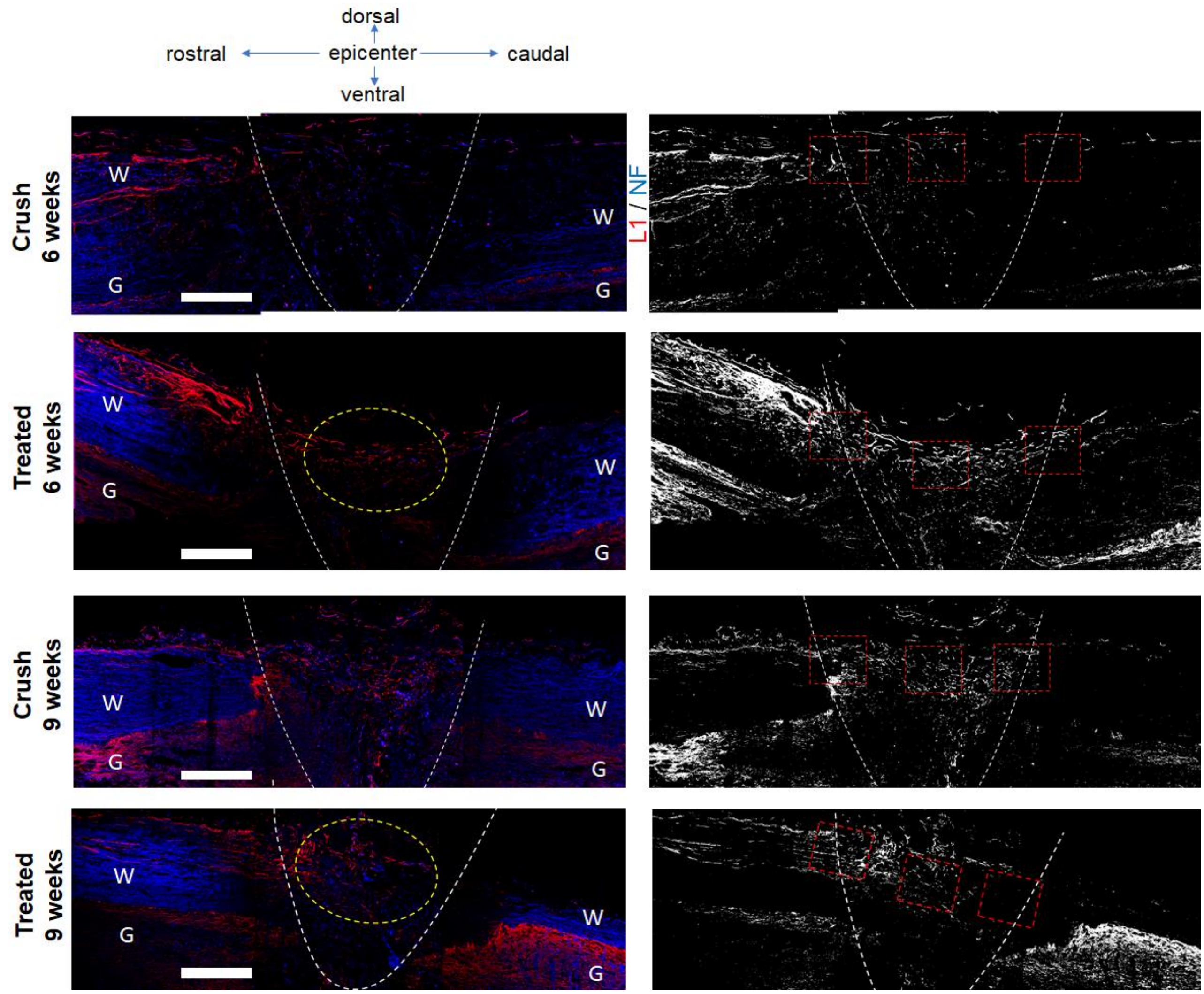

Supplementary Figure 7. Quantification of axonal elongation via L1 immunostaining. Left column: Representative fluorescence images of parasagittal sections from the crush and treated groups immunostained for neurofilament heavy chain and L1, 6 and 9 weeks post injury. The approximate lesion boundary is highlighted via a white dashed line. The approximate site of the remaining graft is highlighted via a yellow dashed ellipse. The locations of the white and gray matter sampled by the specific parasagittal section are highlighted as “W” and “G” respectively. Right column: L1 quantification took place at specific locations (red dashed rectangles) that sample either the L1-positive axons in the dorsal column adjacent (caudally or rostrally) to the lesion boundary, or in the lesion epicenter. Scale bars, 200 μm.

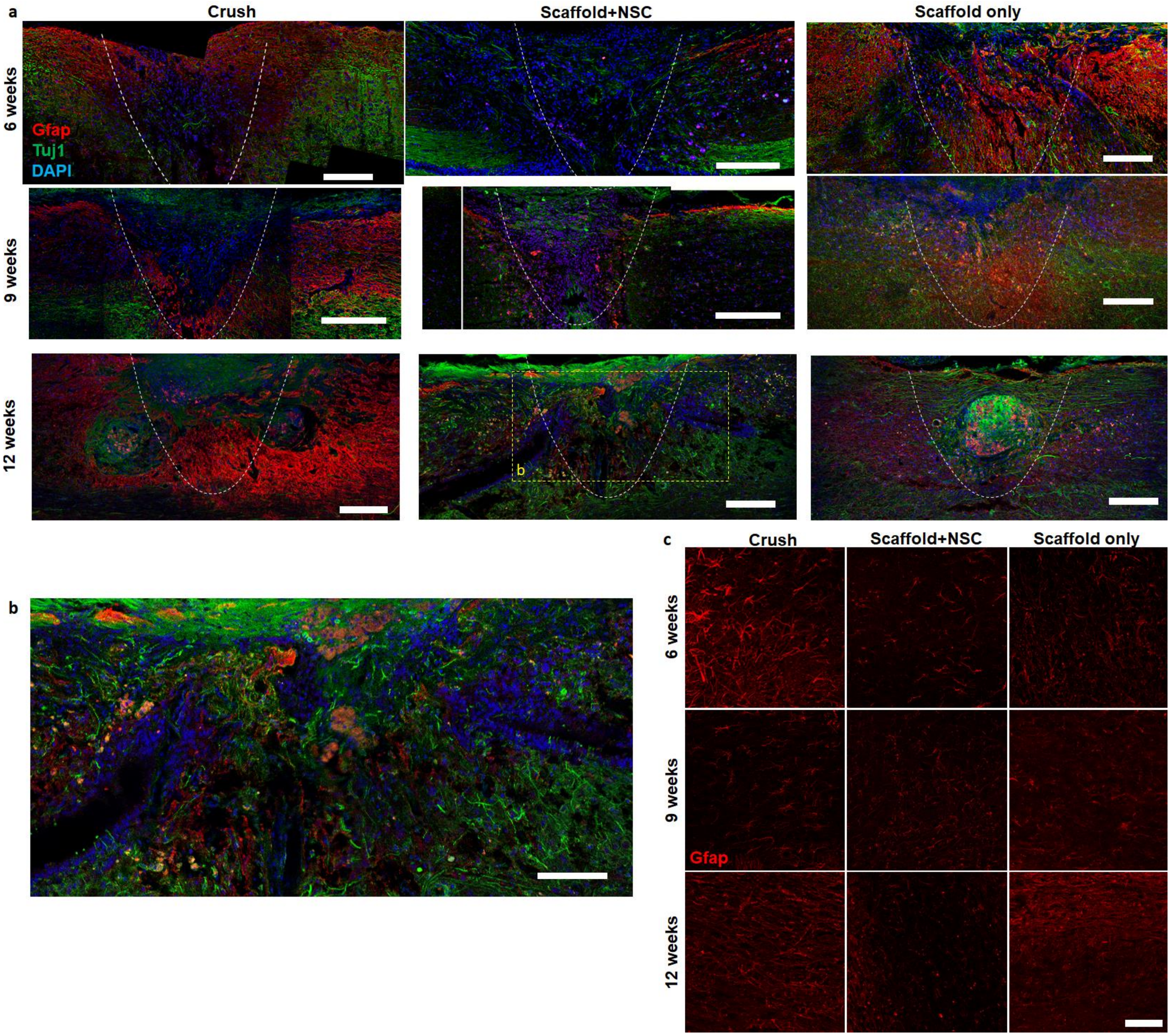

Supplementary Figure 8. Porous collagen scaffolds seeded with embryonic NSCs reduced astrogliosis at the lesion site after dorsal column crush. (**a**) Fluorescence imaging of parasagittal sections stained for Tuj1 and GFAP at the lesion, 6, 9 and 12 weeks post-injury in the "crush", "scaffold+NSC" and "scaffold only" group. The approximate lesion boundary is shown using dashed lines. Bars: 200 μm. (**b**) High-magnification of the region shown in (a) highlights a large number of Tuj1$^+$ axons in the lesion site 12 weeks post injury. Scale bar, 100 μm. (**c**) High-magnification fluorescence images of GFAP$^+$ astrocytes along the approximate lesion boundary at 6 and 9 weeks post injury. Scale bars, 30 μm.

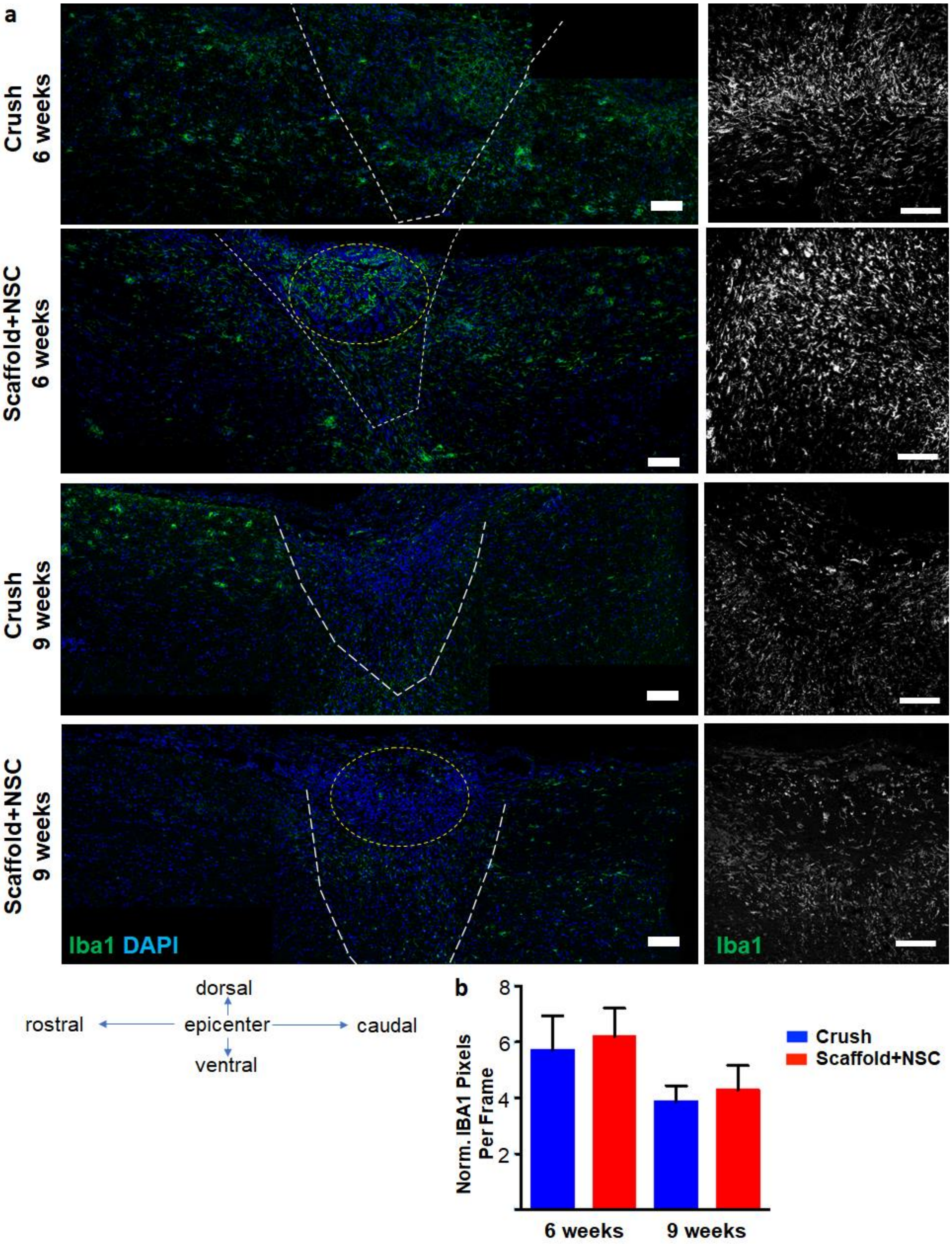

Supplementary Figure 9. Quantification of microgliosis via IBA1 immunostaining. (**a**) Left column: Representative fluorescence images of parasagittal sections from the crush and treated groups immunostained for IBA1, 6 and 9 weeks post injury. The approximate lesion boundary is highlighted via a white dashed line. The approximate site of the remaining graft is highlighted via a yellow dashed ellipse.

Right column: Thresholded IBA1 images at the lesion epicenter utilized for IBA1 quantification. Scale bars, 100 µm (**b**) Quantification of normalized fraction of $IBA1^+$ pixels in the crush and treated groups at 6 and 9 weeks post injury. Results are normalized with respect to the uninjured control group and are presented as mean ± s.e.m. Crush and treated groups: n = 3 to 5.

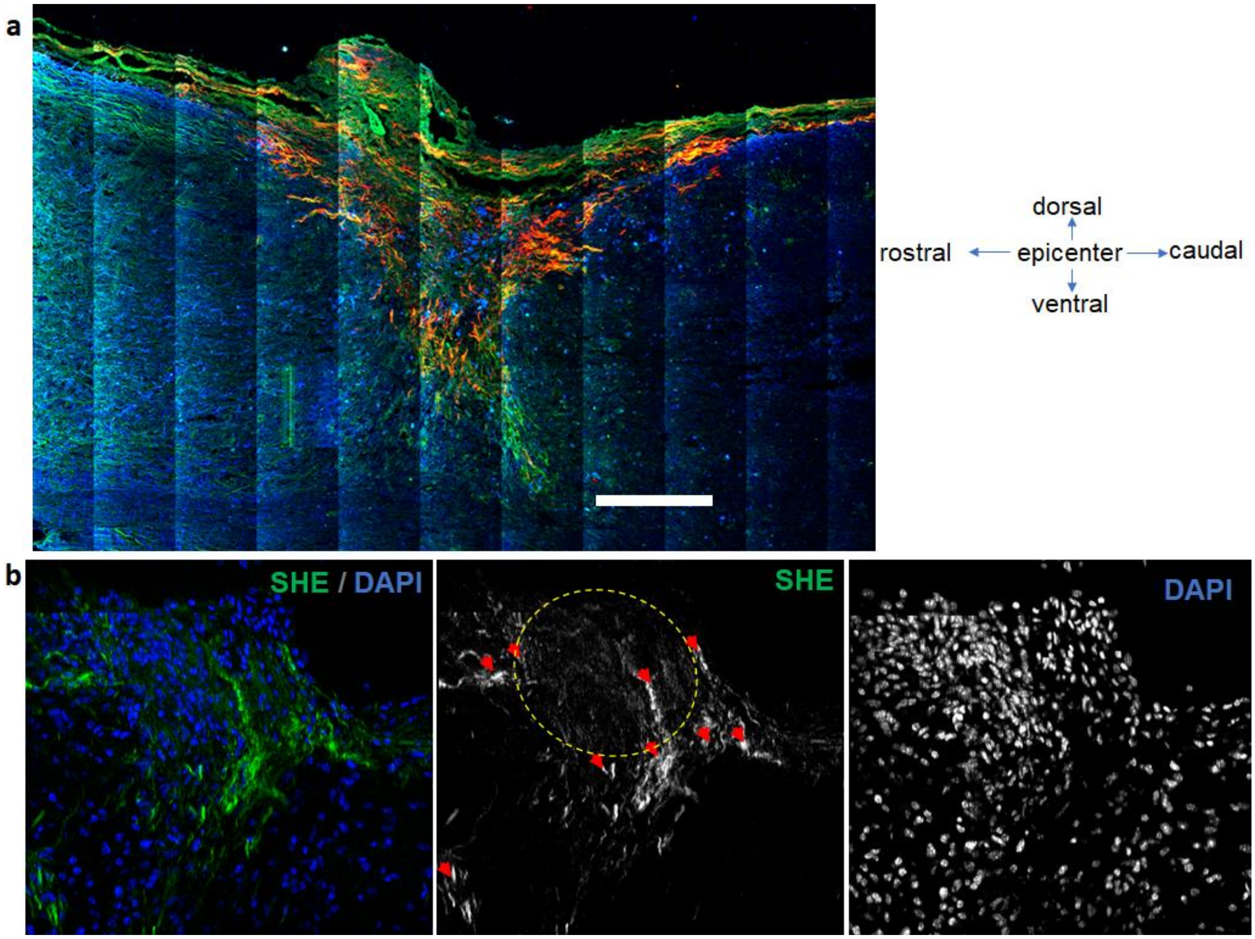

Supplementary Figure 10. Collagen scar formation at the lesion site. (**a**) Representative multiphoton image of a parasagittal section at the lesion site, crush animal group, 9 weeks post-injury. Red: Second harmonic emission (SHE, scar collagen). Blue: GFAP. Green: DAPI, Tuj1 and tissue autofluorescence. Scale bar, 200 µm. (**b**) Representative multiphoton image of a parasagittal section at the lesion site, treated animal group, 9 weeks post-injury. The approximate site of the remaining scaffold (evident by its low SHE) is highlighted via a yellow dashed ellipse. Newly-synthesized scar collagen fibers at the lesion boundary are highlighted using red arrow heads. Scale bar, 200 µm.

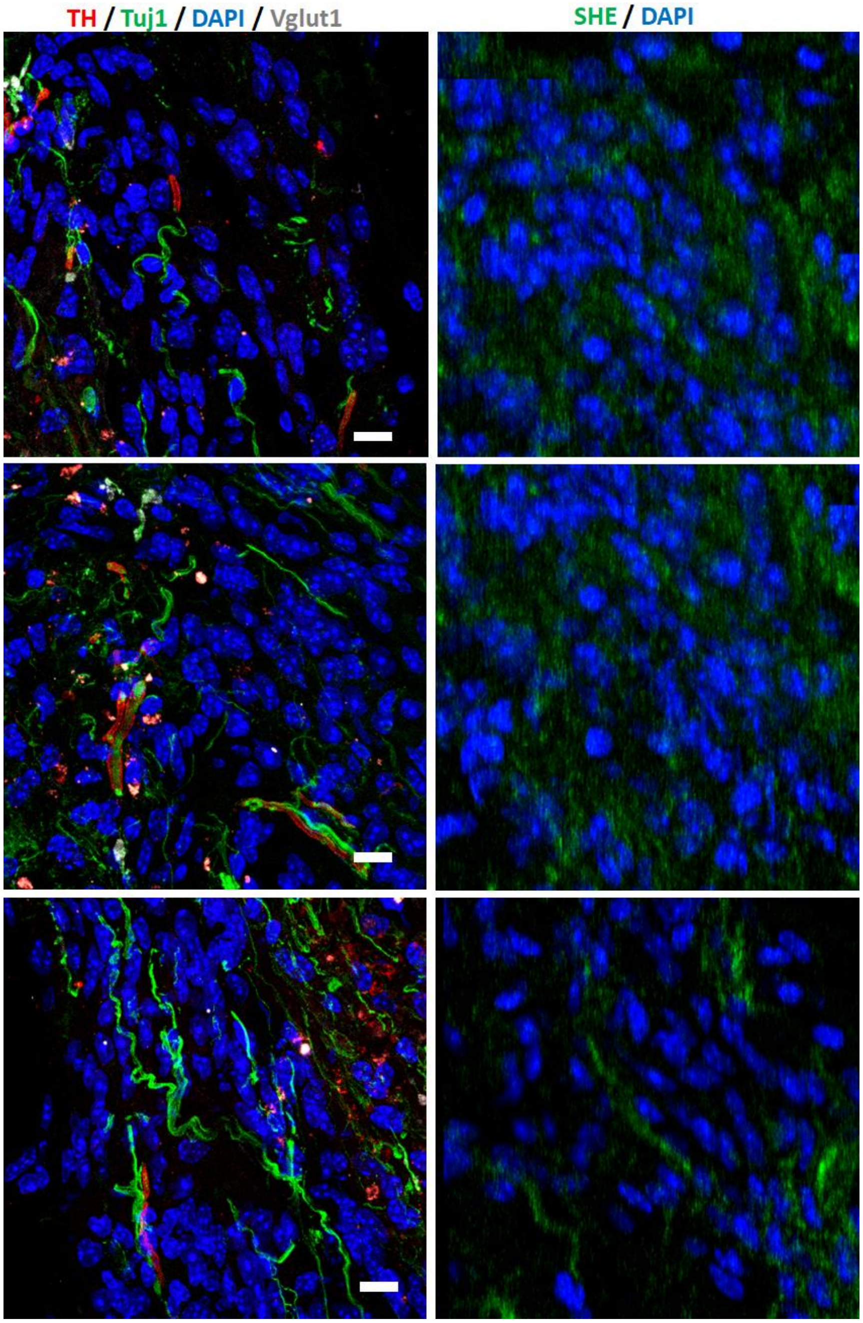

Supplementary Figure 11. Identification of glutamatergic and dopaminergic axons inside the NSC-derived graft. Immunofluorescence images of parasagittal sections acquired inside the graft, at 6 weeks post-injury. Left: sections immunostained for TH (dopaminergic neurons), Vglut1 (glutamatergic neurons) and Tuj1. Right: SHE of residual scaffold collagen in the same locations. Scale bars, 10 μm.

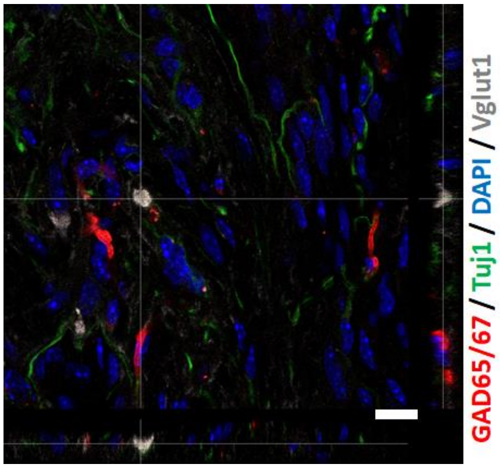

Supplementary Figure 12. Identification of glutamatergic and GABAergic axons inside the NSC-seeded PCS graft. Immunofluorescence image of a parasagittal section acquired inside the graft immunostained for GAD65/67 (Gabaergic neurons), Vglut1 (glutamatergic neurons) and Tuj1, 6 weeks post-injury. Scale bar, 10 μm.
